# Single cell atlas of the comorbidity mechanism between chronic obstructive pulmonary disease and lung adenocarcinoma: a study of multi-omics combined analysis

**DOI:** 10.7717/peerj.20672

**Published:** 2026-02-06

**Authors:** Meng Li, Binyu Wang, Qian Peng, Weiyun Shen, Danfei Shi, Xinmin Li, Daojun Yu, Yong Li

**Affiliations:** 1 Department of Clinical Laboratory, First Affiliated Hospital of Huzhou University, Huzhou, Zhejiang, China; 2 Central Laboratory, First Affiliated Hospital of Huzhou University, Huzhou, Zhejiang, China; 3 Department of Pathology, First Affiliated Hospital of Huzhou University, Huzhou, Zhejiang, China; 4 Chongqing Hospital of Traditional Chinese Medicine, Chongqing, China; 5 Department of Clinical Laboratory, The Fourth Clinical School of Zhejiang University of Traditional Chinese Medicine (Hangzhou First People’s Hospital), Hangzhou, Zhejiang, China

**Keywords:** LUAD, COPD, Single-cell transcriptomics, Immune infiltration, Mendelian randomization, Multi-omics integration

## Abstract

**Purpose:**

Chronic obstructive pulmonary disease (COPD) is a recognized risk factor for lung adenocarcinoma (LUAD), but the molecular mechanisms behind this association are still unclear. This study aims to reveal shared key genes and pathways involved in both COPD and LUAD, and identify potential biomarkers and therapeutic targets.

**Methods:**

Two-sample Mendelian randomization (MR) analysis using Genome-Wide Association Study (GWAS) data was performed to evaluate genetic causality. Differential expression analysis was performed on GSE76925 (COPD) and GSE116959 (LUAD), followed by LASSO regression, enrichment analysis, immune cell infiltration analysis, RNA modification analysis, competing endogenous RNA (ceRNA) network construction, and drug sensitivity prediction. Single-cell RNA sequencing data (GSE270667, GSE189357) were used to explore cell-type-specific expression, and qPCR was performed to validate gene expression in patient blood samples.

**Results:**

MR confirmed the genetic relationship between COPD and LUAD. Three key genes (*FCRLA*, *GREM1*, and *MMP9*) were significantly upregulated and involved in immune regulation, extracellular matrix (ECM) remodeling, and the PI3K-Akt signaling pathway. Single-cell analysis revealed that these genes were specifically expressed in B cells, T cells, and monocytes. Multiple omics analyses indicated epigenetic and RNA level regulation. Several candidate drugs have been identified.

**Conclusion:**

*FCRLA, GREM1*, and *MMP9* are inflammation-associated genes that may link the pathobiology of COPD and LUAD, and serve as valuable biomarkers with therapeutic potential in high-risk populations.

## Introduction

Chronic obstructive pulmonary disease (COPD) and lung adenocarcinoma (LUAD) are two major global health burdens with high incidence rates and mortality (Li & Liu, 2024; Shakeel et al., 2023; Hoang & Landi, 2022). Previous epidemiological studies have shown that COPD patients have a significantly increased risk of developing LUAD, indicating that there may be a common biological mechanism between these two diseases; however, the underlying genetic and epigenetic drivers of their comorbidity have not been explored to a large extent (Cazzola et al., 2023; Jiang et al., 2025). Beyond epidemiological association, these two diseases share common biological underpinnings, notably chronic inflammation, oxidative stress, and tissue remodeling processes, which may create a permissive microenvironment for lung carcinogenesis.

Mendelian randomization (MR) is a powerful method that infers potential relationships. It uses genetic variation as an instrumental variable (Faivre et al., 2024). However, existing MR studies on the association between COPD-LUAD lack comprehensive validation of core MR hypotheses and multi-omics functional exploration. In addition, the dysregulated immune microenvironment may contain key biomarkers bridging the pathological processes of COPD and LUAD, but these targets have not been systematically identified.

This study comprehensively employed two-sample Mendelian randomization analysis, transcriptome data mining, and single-cell RNA sequencing technology (Zhu et al., 2023) to evaluate the potential relationship between COPD and LUAD from multiple perspectives. We also screened key molecules that may play roles in both diseases. By integrating multiple omics databases such as GEO and TCGA (Lillard & Lillard, 2024), we identified and validated three key genes—*FCRLA*, *GREM1*, and *MMP9—*on multiple platforms. These genes are involved in immune regulation, extracellular matrix remodeling, and inflammatory response (Wang et al., 2022a), biological mechanisms that play important roles in the pathogenesis of COPD and LUAD.

The aim of this study is to reveal the potential pathogenic relationship between COPD and LUAD, and to explore in depth the biological significance of *FCRLA*, *GREM1*, and *MMP9* in both diseases. By integrating genetics, machine learning, immune cell infiltration analysis, and epigenetic features, we comprehensively analyzed the shared molecular mechanisms between COPD and LUAD, providing new theoretical basis and potential targets for early screening and precision treatment of LUAD in patients with COPD.

**Figure 1:**
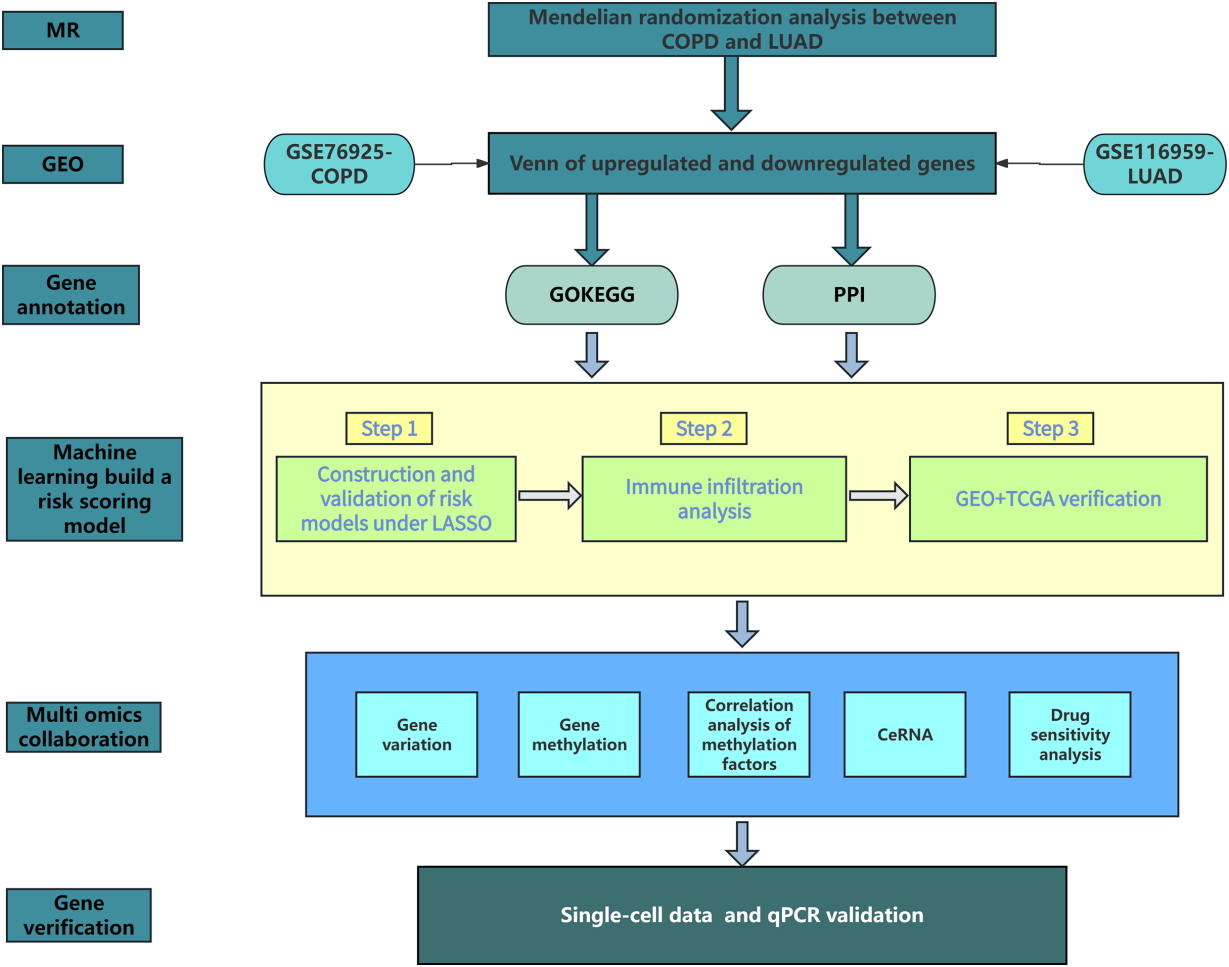
Research workflow.

Figure 1 shows our research workflow.

## Materials and Methods

### Mendelian analysis between COPD and LUAD

We used the TwoSampleMR R package (v0.6.0) to build a two-sample MR framework (R version 4.2.1) (Sollis et al., 2023). The exposure was COPD (Genome-Wide Association Study (GWAS) ID: ebi-a-GCST90018807), and the outcome was LUAD (GWAS ID: ebi-a-GCST004744). The single nucleotide polymorphism (SNP) screening strategy is to select independent variants with genomic significance (*P* < 5 × 10^−8^) in COPD GWAS, and use linkage disequilibrium (LD) clustering (r^2^ < 0.001, window 10,000 kb) to eliminate linkage disequilibrium sites (Liao et al., 2024). The extracted instrumental variables (IVs) were clustered on an external LD reference panel (1,000 Genomes, EUR). If a SNP is missing in the outcome GWAS, it will be replaced by LD proxy (r^2^ ≥ 0.8); If it cannot be replaced, discard it (Liao et al., 2024). We evaluated the IV intensity (calculated F-statistics for each SNP) and excluded weak instrumental variables with F < 10. The main effect estimation was performed using the inverse variance weighted (IVW) method, supplemented by MR Egger, Weighted Median (WM), Simple Mode, and Mendelian Randomization Pleiotropy RESidual Sum and Outlier (MR-PRESSO) as sensitivity analyses. All single SNP and multiple comparison tests report the original *P*-value and Benjamini Hochberg corrected q-value (if multiple comparison is performed) (Mohus et al., 2022).

### Data sources

Data related to “LUAD” and “COPD” were queried separately in the GEO database, resulting in the selection of two datasets: GSE116959 (Ashique et al., 2025) and GSE76925 (Myszko et al., 2025). In addition, we searched the relevant single cell data set, and finally obtained GSE270667 and GSE189357 (Feng et al., 2024).

Details of each dataset are as follows:

GSE116959 data set (Ashique et al., 2025): Platform: GPL17077 (Agilent-039494 SurePrint G3 Human GE v2 8 60 K Microarray Chip 039381), specimens: 68 independent samples, 57 LUAD (T), and 11 healthy lungs (N).
GSE76925 data set (Myszko et al., 2025): Platform: GPL10558 (Illumina HumanHT-12 V4.0 Expression chip),
Specimens: 111 COPD cases and 40 control smokers.
GSE270667 data set: Platform: GPL16791 (Illumina HiSeq 2500 *Homo sapiens*), Specimens: six COPD cases and six control cases.
GSE189357 data set (Feng et al., 2024): Platform: GPL24676 (Illumina NovaSeq 6000 *Homo sapiens*), Specimens: Nine resected samples (TD1-9, three each from AIS, MIA and IAC cases) from nine treatment-naïve patients.

The datasets offer valuable information for gene expression analysis in LUAD and COPD, serving as a comprehensive resource for future studies.

### DEG data handling

GEO data (Barrett et al., 2012) were extracted using the R-based web application GEO2R. Differential expression analysis was then performed using the limma package (version 3.52.4). Probes were annotated as gene symbols, and probes with *P*.adj < 0.05 and |logFC| ≥ 1 are defined as significant differentially expressed genes (DEGs) (Reis-de-Oliveira et al., 2024). Subsequently, volcano plots were generated using the R package “ggplot2” (version 3.3.6) and a Venn diagram was created using VENNY (version 2.1) to visually represent the identified DEGs (Zhang et al., 2024b).

### KEGG and GO enrichment analysis of DEGs

To clarify the functional significance of overlapping DEGs, we performed Gene Ontology (GO) annotation and Kyoto Encyclopedia of Genes and Genomes (KEGG) pathway analysis (Zhang et al., 2024a). Analysis was performed using the Annotation, Visualization and Integrated Discovery Database (DAVID) tool (version 6.7) (Zhang et al., 2024a). In our study, statistical significance was determined using a corrected *P* < 0.05 (Zhang et al., 2024b).

### The PPI network of the DEG

In our study, a protein–protein interaction (PPI) network was constructed using the STRING database and visualized with Cytoscape (version 3.7.2) (Doncheva et al., 2022). Additionally, the R package “ggplot2” (version 3.3.6) was employed to create string plots of DEGs in LUAD (Zhang et al., 2024b).

### Machine learning build a risk scoring model

We used LASSO regression and the glmnet R package (v4.1.8) to identify key biomarkers and construct a risk scoring model (Xie, Shi & Han, 2023). The optimal penalty parameter λ was determined through 10-fold cross-validation, selecting the value that minimizes binomial bias (Zhang et al., 2024b). The final risk score is calculated as follows: risk score = (−0.1691 × *FCRLA* expression) + (0.0629 × *GREM1* expression) + (0.0124 × *MMP9* expression).

### Analysis of the infiltration of the immune cells

The ssGSEA algorithm (Huang et al., 2021) was applied to the TCGA-LUAD (lung adenocarcinoma) project to identify significant correlations between the target gene and various immune cells. All analyses and visualizations were performed using R software (version 4.2.1). Based on markers for 24 immune cells provided by the Immunity article (Hoekstra et al., 2024), expression data were obtained by downloading RNAseq data processed by STAR from the TCGA-LUAD project in TPM format, along with clinical data (Roh et al., 2022).

### The TCGA and GEO databases verified three target genes

We acquired and organized the data from the TCGA-LUAD project, obtained from the TCGA database (Captier et al., 2025). Subsequently, we analyzed the expression levels and receiver operating characteristic (ROC) curves of the target genes in lung adenocarcinoma using both the TCGA-LUAD and GSE76925 datasets. This analysis was carried out utilizing the R programming language software (version 4.2.1). Data was analyzed by ROC using pROC package [1.18.0] and the results were visualized using ggplot2 [3.3.6] (Zhang et al., 2024b). We acquired pre-processed RNA-seq data (in TPM format) for the TCGA-LUAD cohort from the GDC data portal and collated it for analysis. No realignment was performed; the data were used as provided by TCGA (Roh et al., 2022).

### Analysis of genetic alterations

The cBioPortal website is an integrated platform for analyzing and visualizing cancer genomics data (de Bruijn et al., 2023). We used the database from UCSC Xena and the International Cancer Genome Consortium (ICGC) (Wang et al., 2022b). The “Cancer Type Summary” module was used to investigate the mutation landscape of *FCRLA, GREM1, and MMP9*, including mutation types, copy number alterations (CNA), and mutation frequencies.

### DNA methylation and mRNA modification

UALCAN (Rubey et al., 2025) was used to explore *FCRLA* in normal and pan-cancer tissues, as well as *GREM1* and promoter DNA methylation levels of *MMP9*. This value represents the DNA methylation level, with low methylation levels ranging from 0.25 to 0.30, and high methylation levels ranging from 0.7 to 0.5. The DNA methylation profiles of *FCRLA*, *GREM1*, and *MMP9* in LUAD were obtained from the MethSurv database (Modhukur et al., 2018). A useful bioinformatic online tool for mRNA modification analysis of n1-methyladenosine (m1A) and 5-methylcytosine (m-5c) n6-methyladenosine (m6A) in pan-cancer tissues is SangerBox3.0 (Chen et al., 2024).

### Construction of the ceRNA regulatory network

Four online miRNA prediction databases—miRWalk (Sticht et al., 2018), miRDB (Chen & Wang, 2020), ENCORI (Cui et al., 2024), and TargetScan (Mon-López & Tejero-González, 2019)—were used to predict the target miRNAs of *FCRLA, GREM1*, and *MMP9*. Define miRNAs retrieved from at least three databases as targeted miRNAs, then construct an lncRNA miRNA interaction network using the StarBase2.0 (Li et al., 2014) database, and further analyze the regulatory relationship of lncRNA miRNA mRNA by combining mRNA data. The visualization of the lncRNA-miRNA-mRNA interactions was separately performed using Cytoscape (version 3.7.2) (Zhang et al., 2024a). Additionally, Sankey plots were generated using the R package “ggplot2” (version 3.3.6) (Zhang et al., 2024b).

### Analysis of drug response

GSCALite (Wei et al., 2025) was used to analyze the integrated mRNA expression, mutation, immune infiltration, and methylation of the TCGA data sets derived from GDSC and CRTP (Wu et al., 2024). Using data from GDSC and CRTP, we analyzed sensitive drugs for *FCRLA, GREM1, and MMP9*, and identified Food and Drug Administration (FDA)-approved chemotherapy drugs associated with their expression in the Drug Bank (Knox et al., 2024). The results were visualized using Cytoscape (version 3.7.2) (Zhang et al., 2024a).

### Single cell data validation

This study involved two datasets, GSE270667 and GSE189357. All patients had not received treatment. The collected lung adenocarcinoma samples were analyzed by droplet based single cell RNA sequencing technology (Myszko et al., 2025). The cell suspension was processed by microfluidic chip to ensure the efficiency of cell capture and RNA extraction (Feng et al., 2024). The cell suspension was processed by a microfluidic chip to ensure efficient cell capture and RNA extraction (Feng et al., 2024). The Seurat package (v4.2.1) (Arbatsky et al., 2025) was used to process single-cell data. Cells with fewer than 200 genes or mitochondrial reads exceeding 10% were filtered out. Data were normalized using the SCTransform method. Principal component analysis (PCA) and uniform manifold approximation and projection (UMAP) were used for dimensionality reduction (Haggar et al., 2024). The cell cluster resolution for GSE270667 and GSE189357 was set to 0.8, and clusters were annotated based on typical marker genes.

### Supplementary validation by qPCR

In this study, the expression levels of *FCRLA*, *GREM1*, and *MMP9* in COPD and LUAD were determined by quantitative polymerase chain reaction (qPCR). We recruited 12 patients with LUAD and 12 patients with COPD who were hospitalized from January 1, 2024 to January 31, 2024, and included 12 healthy people who underwent routine health examination in the same period as the control group. The inclusion criteria for patients with lung cancer were: (1) age ≤ 90 years old; (2) having no history of chemoradiotherapy; (3) not experiencing fever or infection within 3 months prior to blood collection; and (4) having no history of blood transfusion (Zhang et al., 2024b).

After RNA extraction from human whole blood using the Blood Genome Purification Kit (Genefist, Shanghai, China), RNA was converted to cDNA using the high-volume cDNA Reverse Transcription Kit (Takara Biotech (Beijing), China) according to the manufacturer’s instructions (Cai et al., 2024). RT-qPCR assays were performed in 20 μL of the reaction mixture containing 10 μL SYBR green master mixture, 0.8 μL forward primer, 0.8 μL reverse primer, 0.4 μL ROX reference dye, 2 μL cDNA and 6 μL nuclease-free water. The thermal cycling conditions are as follows: 95 °C for 30 s, followed by 40 cycles of 95 °C for 5 s and 60 °C for 34 s. The expression level of each gene was analyzed using the 2^−ΔΔCt^ method (Shi et al., 2024). The sequence of the primer pair is as follows:

*FCRLA*: forward primer, 5′-CACACGGAGGATGACTTGACTGATG-3′, and reverse primer, 5′-TGGCTTGGCTGGACCTTGGAG-3′.

*GREM1*: forward primer, 5′-TCGCACCATCATCAACCGCTTC-3′, and reverse primer, 5′-CAGAAGGAGCAGGACTGAAAGGAAC-3′.

*MMP9*: forward primer, 5′-CCCTGGTCCTGGTGCTCCTG-3′, and reverse primer, 5′-CTGCCTGTCGGTGAGATTGGTTC-3′.

*GAPDH* is used as a standardized endogenous control. The primer sequence for GAPDH is: forward 5′-GGAGCGAGATCCCTCCAAAAT-3′, reverse 5′-GGCTGTTGTCATACTTCTCATGG-3′.

### Population

The study protocol was approved by the Medical Ethics Committee of the Medical Research and Clinical Trial Ethics Committee of Huzhou First People’s Hospital (Approval Number: 2023KYLL014). All patients participating in this study provided written informed consent.

### Statistical analysis

The statistical analysis in this study was performed using the online database described above and in R (4.2.1), as described above. Differences between groups were evaluated using Student’s t-test, and results are presented as mean ± standard deviation (SD). Statistical significance was reported as *P < 0.05, **P < 0.01, ***P < 0.001.

## Result

### Univariate Mendelian randomization analysis of COPD and LUAD

**Figure 2:**
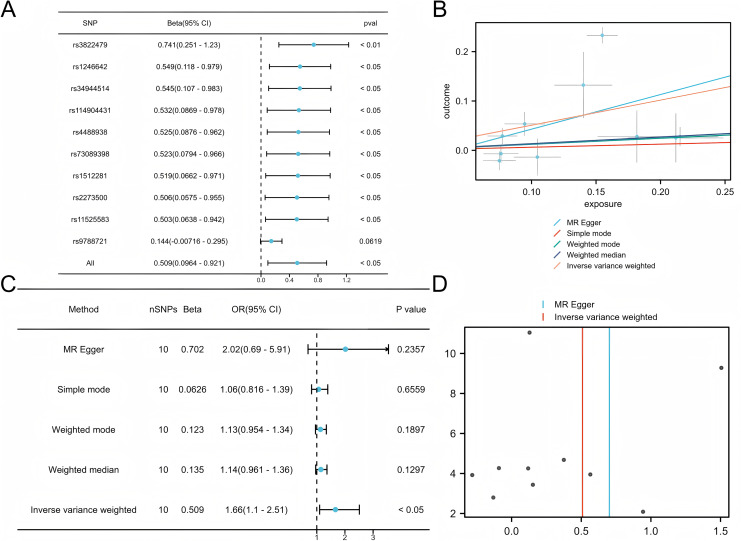
Mendelian analysis of COPD and LUAD. (A) Leave one out for the Mendelian analysis of COPD and LUAD, forest plot. (B) Scatter plot of COPD *vs* LUAD. (C) Forest plot of the Mendelian analysis of COPD and LUAD. (D) Funnel plot of the COPD *vs* LUAD Mendelian analysis.

Five commonly used MR methods (IVW, WM, MR-Egger, Simple Mode, and MR-PRESSO) were used to assess the association between COPD and LUAD (Lu et al., 2025). The primary analysis using the IVW method demonstrated a significant causal inference between COPD and LUAD (*P* < 0.05) with consistent directionality, suggesting that COPD is a potential risk factor for LUAD (Fig. 2). Among the ten SNPs identified by the TwoSampleMR package, nine showed significant associations (*P* < 0.05), further supporting the robustness of the potential inference.

### Identification of the differentially expressed genes

**Figure 3:**
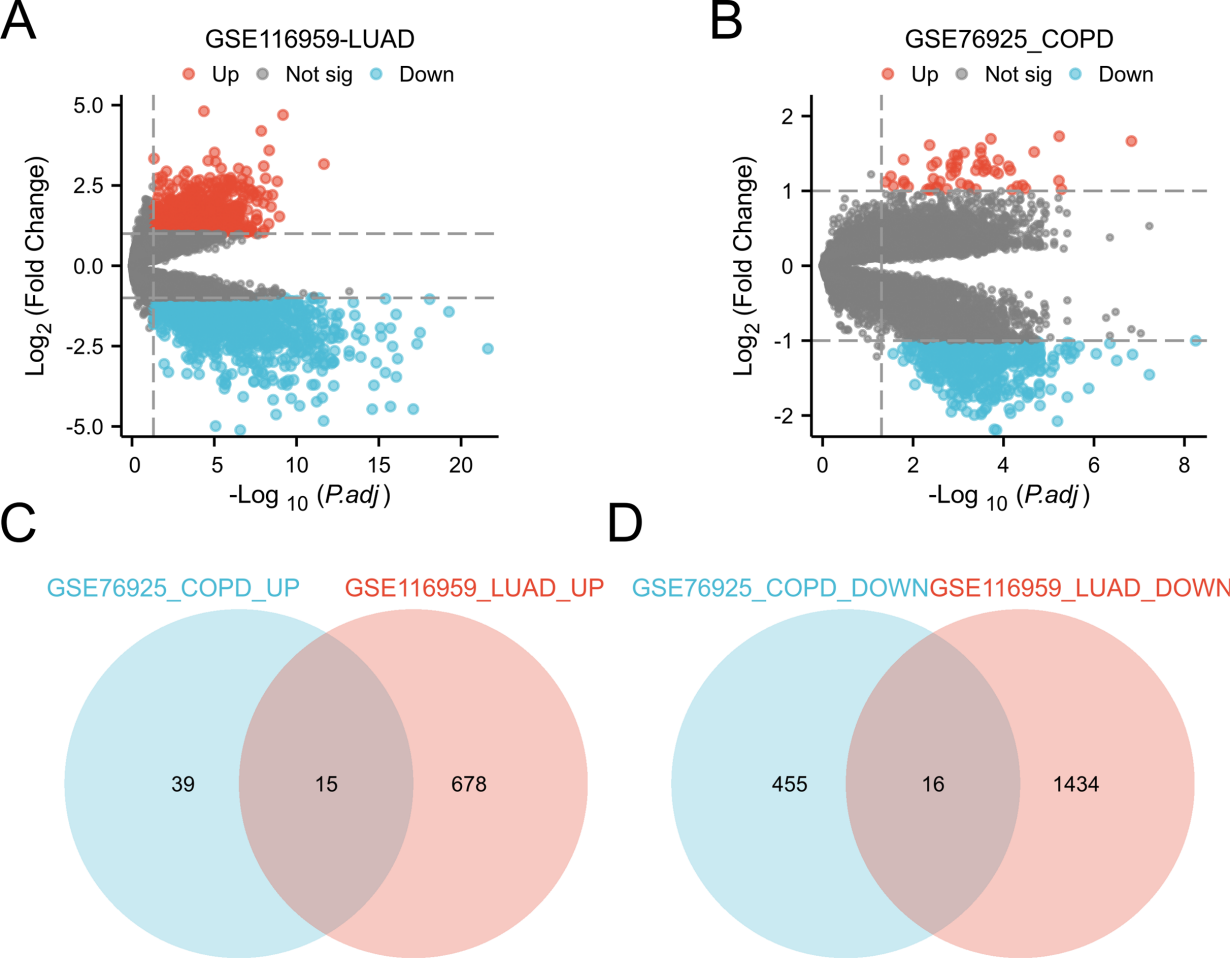
Identification of DEGs in the two GEO datasets. (A) Volcano plot of DEGs for expression differences between lung adenocarcinoma specimens and normal lung specimens in the GSE116959 dataset. (B) Volcano plot of DEGs with variable expression between COPD specimens and normal lung specimens in dataset GSE76925. (C) Venn plot highlighting 15 overlapping upregulated DEGs in the GSE116959 and GSE76925 datasets. (D) Venn plots showing the 16 overlapping downregulated DEGs in the GSE116959 and GSE76925 datasets.

Differential expression analysis was conducted by comparing LUAD tissue and normal lung tissue in the GSE116959 dataset, as well as COPD tissue and control smoker tissue in the GSE76925 dataset (Ashique et al., 2025; Myszko et al., 2025). Genes with *P*.adj < 0.05 and |logFC| ≥ 1 are considered significant. This identified 31 common DEGs in two diseases, including 15 upregulated genes (*CD19, COL10A1, COMP, FCRLA, POU2AF1, MMP11, VPREB3, BLK, CXCR5, MMP9, SPP1, CXCL13, GREM1, XAGE1A, SRPX2*) and 16 downregulated genes (*ID4, HMCN1, FRY, ARHGAP18, CCDC102B, TYRP1, MEOX2, FCGR3A, BCHE, ECHDC1, NEDD9, GIMAP1, METTL7A, HPGDS, ATP13A4, PTPLAD2*) (Fig. 3). These genes show significant expression differences in COPD and LUAD, and may be involved in the common mechanisms of these two diseases.

### KEGG and GO enrichment analysis of the DEGs

Through GO and KEGG analysis (Zhang et al., 2024a), it was found that upregulated genes are mainly involved in the following biological processes and pathways:

Biological processes (BP): extracellular structural organization, extracellular matrix remodeling.

Cellular component (CC): extracellular matrix with collagen content.

Molecular function (MF): Cytokine binding, bone morphogenetic protein (BMP) binding.

**Figure 4:**
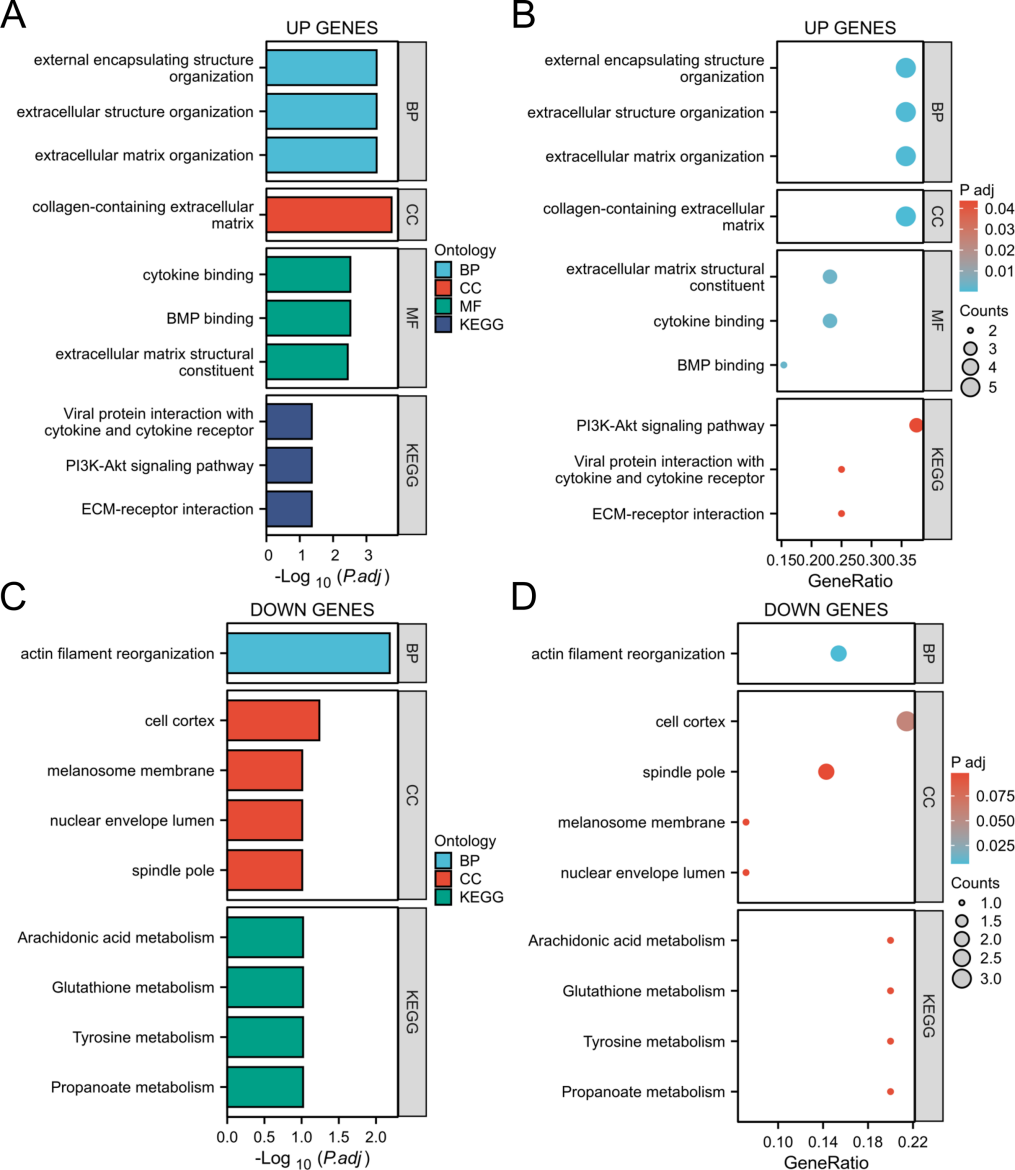
GO/KEGG analysis of the DEGs. (A, B) GO/KEGG analysis of the upregulated genes; (C, D) GO/KEGG analysis of the down-regulated genes.

In addition, KEGG pathway analysis showed that these genes were significantly enriched in the PI3K Akt signaling pathway and extracellular matrix (ECM) receptor interactions, suggesting their key roles in inflammation and tumor microenvironment (Figs. 4A, 4B). The downregulated genes are mainly related to metabolic pathways such as arachidonic acid metabolism and glutathione metabolism, suggesting that they may play an important role in cellular homeostasis (Figs. 4C, 4D).

### The PPI network construction

**Figure 5:**
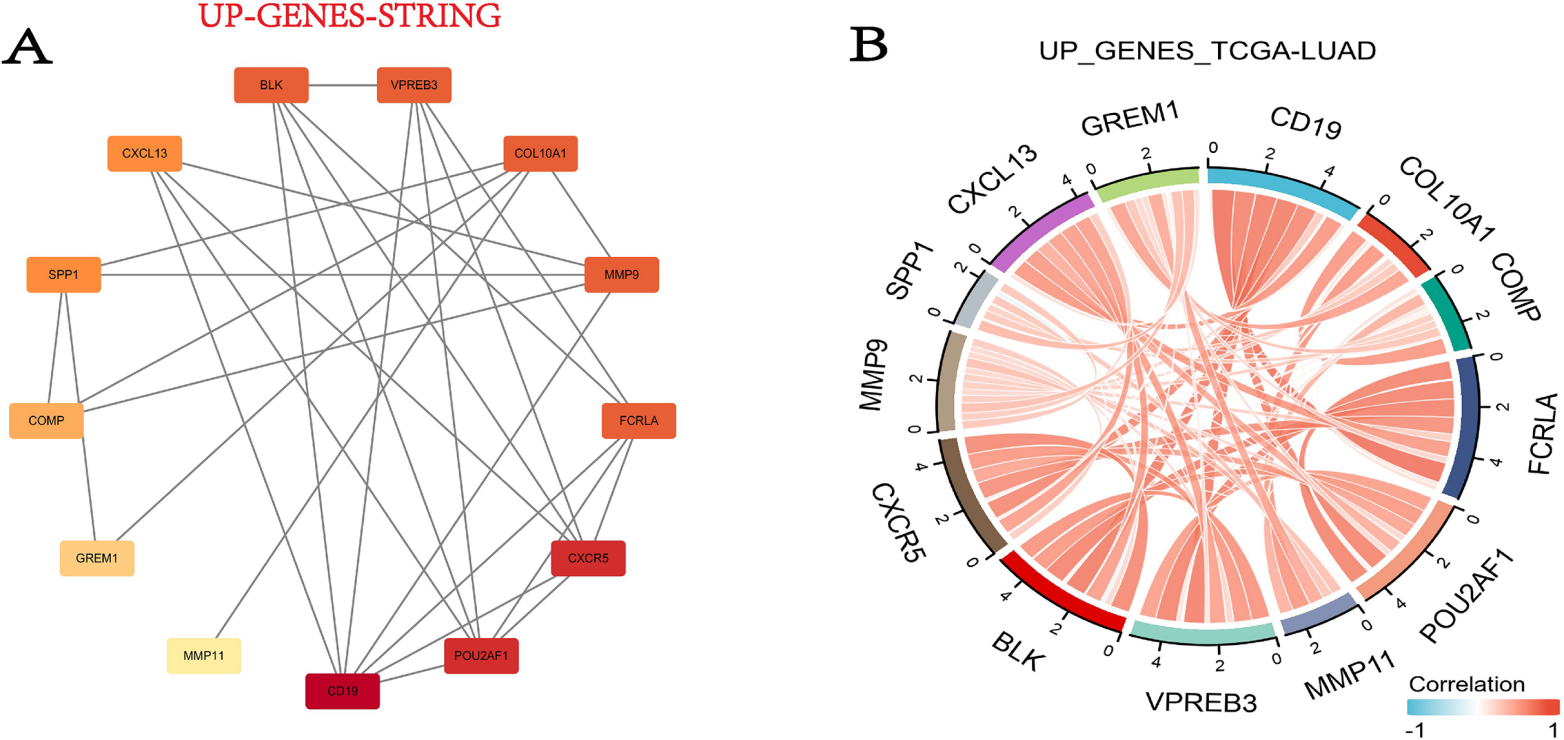
The DEG PPI network. (A) The PPI network map of the upregulated genes. The darker the color, the higher the upregulation. (B) String plot of TCGA-LUAD correlation of upregulated genes.

In this step, we used the STRING database to build the PPI network for DEGs. Fortunately, 13 out of the 15 upregulated genes successfully made the PPI network, while the 16 down-regulated genes were not successful (Fig. 5A). Then we combined the TCGA-LUAD project to produce the correlation string diagram (Fig. 5B). The PPI network reveals strong interactions between upregulated genes, especially those related to the ECM and immune proteins. This suggests their synergistic role in tissue remodeling and immune regulation in COPD and LUAD.

### Target genes were selected by the LASSO analysis and a risk scoring model was built

**Figure 6:**
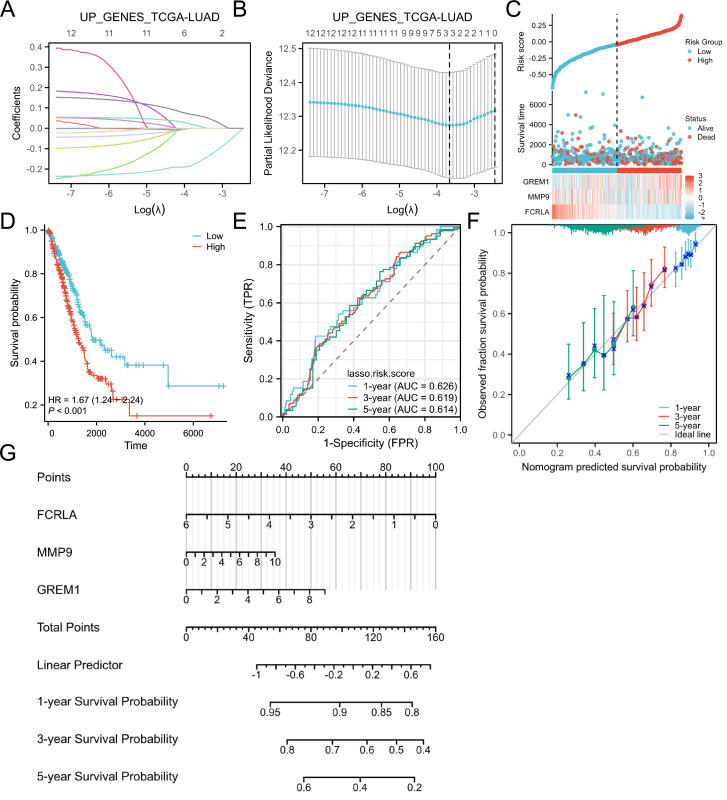
Lasso and risk scoring models. (A) LASSO analysis coefficient changes with the λ parameter; (B) LASSO analysis λ corresponding trend with the number of variables. (C) Risk score, survival time distribution, and gene expression heatmap of relevant genes in the TCGA-LUAD cohort. (D) Kaplan Meier survival analysis of OS between risk groups. (E) ROC curves of risk scoring models for predicting 1, 3, and 5-year OS. (F) Corresponding calibration curve. (E) Prognostic nomogram of 1, 3, and 5-year OS for three genes.

The LASSO regression analysis identified the characteristic genes in the hub genes, namely *FCRLA*, *GREM1* and *MMP9*. These three genes were then incorporated into a nomogram model, the risk score for each patient was calculated. The findings of the study indicated that, these genes are key candidates with important roles in the biological systems studied, particularly in lung adenocarcinoma (Fig. 6).

*FCRLA* is a gene primarily expressed in B cells that regulates immunoglobulin trafficking and may inhibit B cell development and function (Tolnay, 2022). *GREM1* is a chemokine family growth factor essential for embryonic development and tissue formation, with abnormal expression linked to various cancers and congenital malformations (Davis et al., 2015). *MMP9* is a matrix metalloproteinase that degrades the extracellular matrix, influencing cell migration, proliferation, and tissue repair, and is implicated in inflammation, tumor metastasis, and cardiovascular disease (Zhou et al., 2025). Subsequently, these three genes were used for multi-omics validation to elucidate their functional roles and regulatory mechanisms in COPD-LUAD comorbidity.

### Immune infiltration analysis

**Figure 7:**
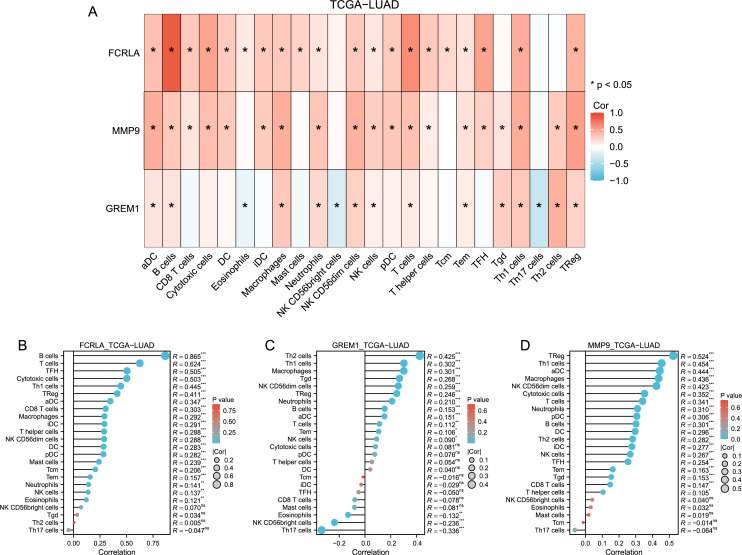
Immune-related infiltration analysis of these three target genes. (A) Heat map of the correlation between the three target genes and each immune cell in TCGA-LUAD (lung adenocarcinoma); (B, C and D) Bar plots of correlation between *FCRLA, GREM1, MMP9* and various immune cells.

Immune cell infiltration analysis revealed that *FCRLA*, *GREM1*, and *MMP9* were significantly correlated with various immune cells, such as CD8+T cells and B cells (Fig. 7). This association suggests that these genes may be involved in the common mechanisms of COPD and LUAD by regulating immune responses in the tumor microenvironment. For example, *FCRLA* is strongly positively correlated with B cell infiltration (r = 0.865, *P* < 0.001), which is consistent with its known role in B cell biology. *MMP9* is associated with monocyte infiltration (r = 0.436, *P* < 0.001), consistent with its role in monocyte derived matrix degradation and inflammation.

### Multidimensional validation of target genes

**Figure 8:**
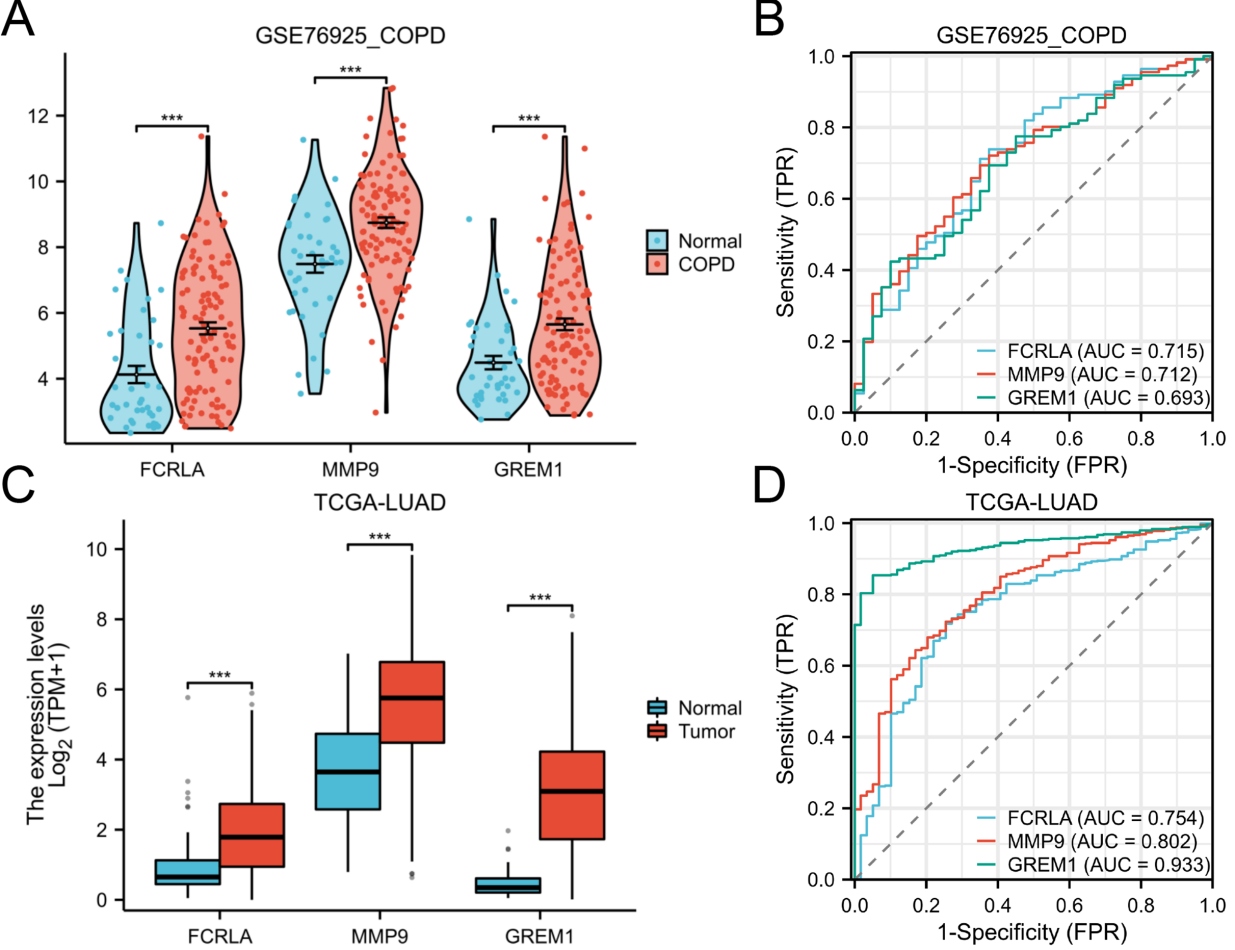
Expression of the three target genes, *FCRLA*, *GREM1*, and *MMP9*, in COPD and lung adenocarcinoma. (A, B) Expression level and ROC curve of these three genes in COPD; (C, D) Expression levels and ROC curves of these three genes in lung adenocarcinoma. Data are presented as mean ± SD. Statistical significance was determined by two-tailed Student’s t-test (***P < 0.001).

The expression patterns and diagnostic performance of *FCRLA*, *GREM1*, and *MMP9* in COPD and LUAD were validated using TCGA and GEO data. The ROC curve shows significant expression differences of these three genes between the disease group and the control group, further supporting their value as potential biomarkers (Fig. 8).

### Analysis of genetic alterations

**Figure 9:**
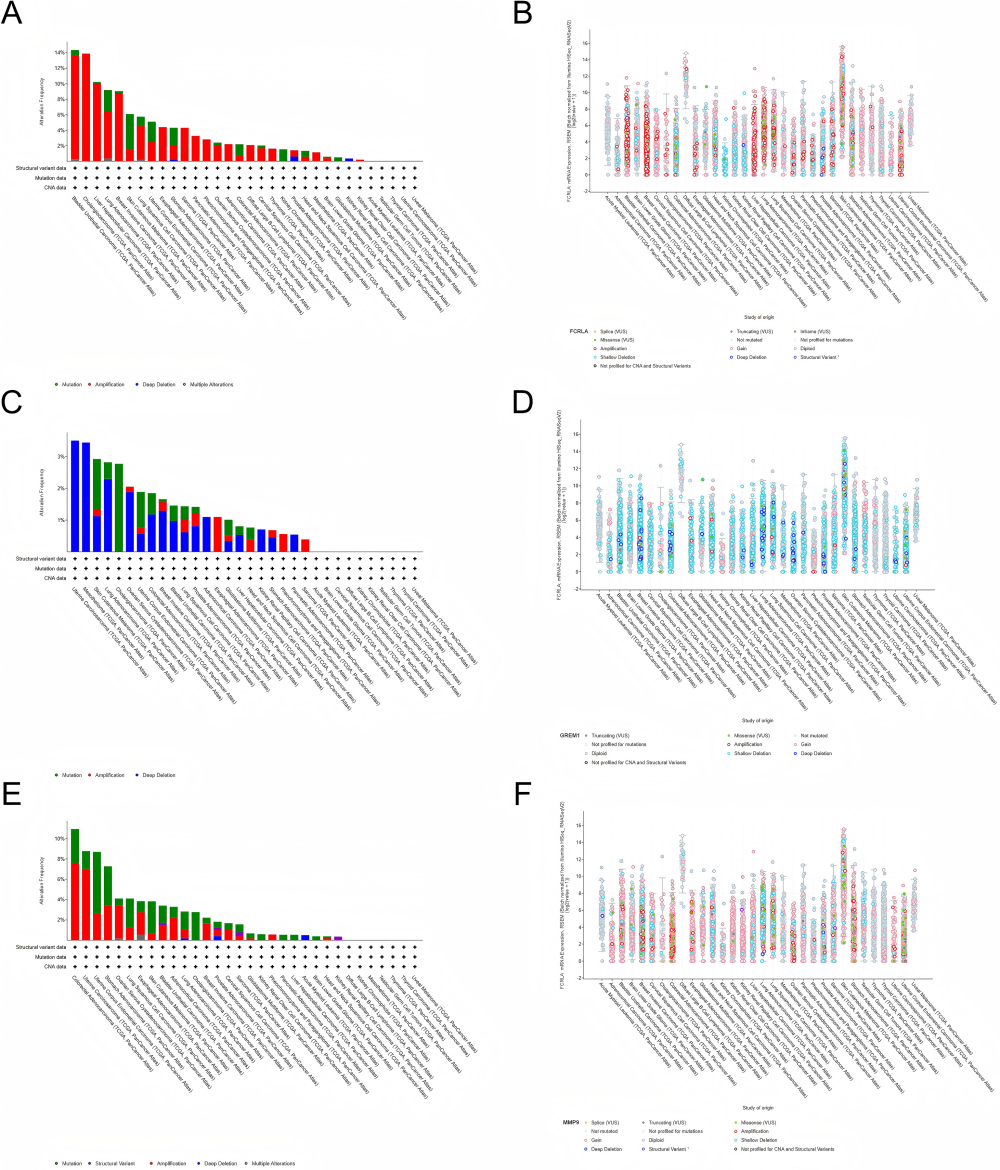
Analysis of the genetic alterations. (A, B) Altered frequency of *FCRLA* mutation type and mRNA expression of putative copy number alteration (CAN) in different cancers; (C, D) altered frequency of *GREM1* mutation type and mRNA expression of putative copy number alteration (CAN) in different cancers; (E, F) altered frequency of *MMP9* mutation types and mRNA expression of putative copy number alteration (CAN) in different cancers.

We investigated the genetic alterations in *FCRLA, GREM1*, and *MMP9* in pan-cancer using cBioPortal (Gneiting & Vogel, 2022). The mutation rates of target genes were observed in all 32 cancers, with the highest mutation rate in bladder urothelial carcinoma (BUC) patients in *FCRLA*, UCS patients in *GREM1* and colorectal carcinoma (CRC) patients in *MMP9*. No elevated mutation rates were observed in several cancers, including uveal melanoma (UM). Subsequently, we investigated the correlation between putative copy number alterations (CNA) in *FCRLA, GREM1*, and *MMP9* and mRNA expression in pan-cancer tissues (Fig. 9).

### Epigenetic alterations in LUAD

**Figure 10:**
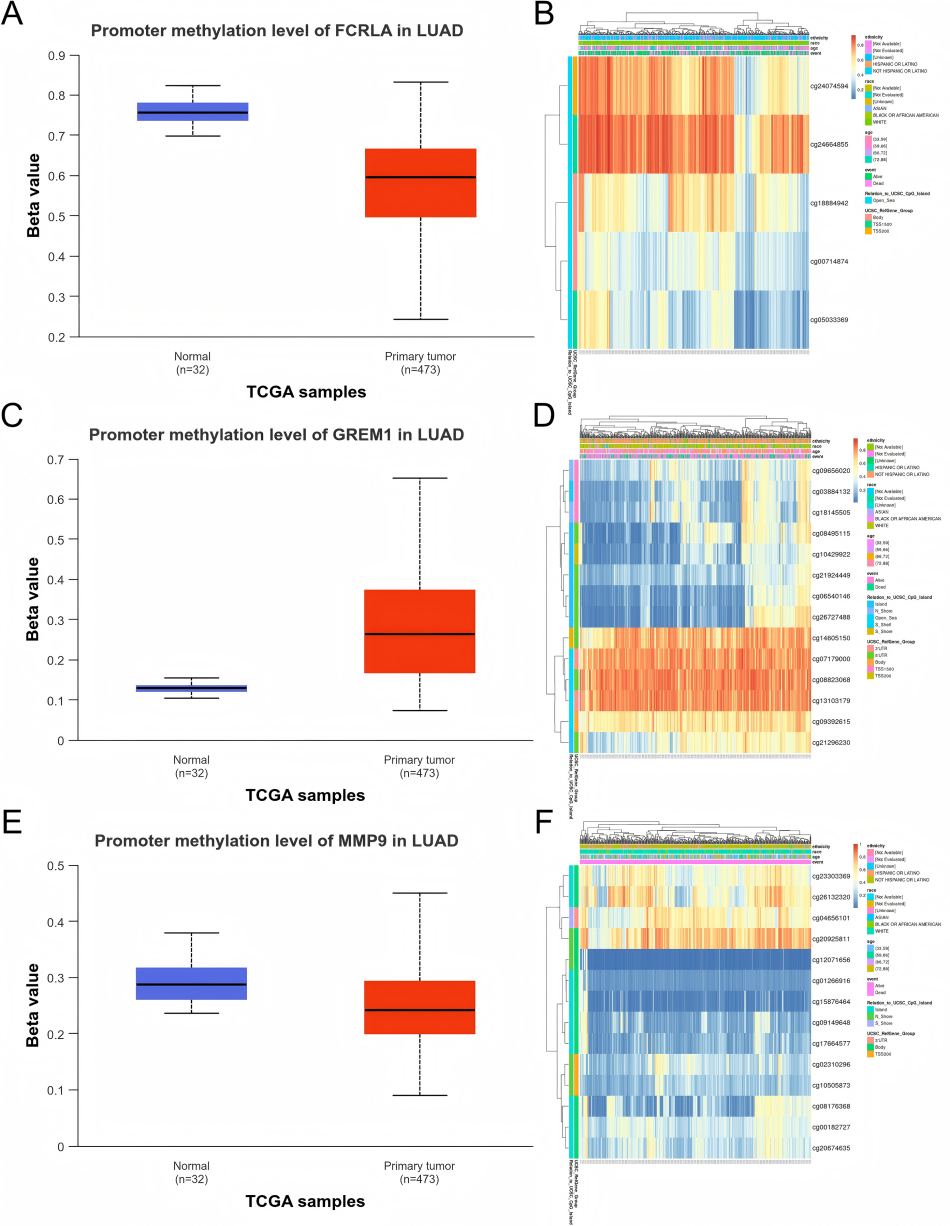
DNA methylation analysis of the target genes. (A) *FCRLA* promoter methylation levels in normal tissues and LUAD tissues; (B) Heatmap of *FCRLA* methylation in LUAD. (C) *GREM1* promoter methylation levels in normal tissues and LUAD tissues; (D) Heatmap of *GREM1* methylation in LUAD. (E) Promoter methylation levels of *MMP9* in normal tissues and LUAD tissues; (F) Heatmap of *MMP9* methylation in LUAD.

We evaluated the promoter DNA methylation levels of *FCRLA*, *GREM1*, and *MMP9* in normal and LUAD tissues. The results showed that *FCRLA* and *MMP9* exhibited significant low methylation status in LUAD tissues, while *GREM1* showed high methylation characteristics. These differences in methylation patterns may regulate the expression of target genes, thereby affecting their functions in LUAD. Through the analysis of the MethSurv database, the key CpG sites of these three genes in LUAD were further identified, providing more evidence for revealing their epigenetic roles (Fig. 10).

**Figure 11:**
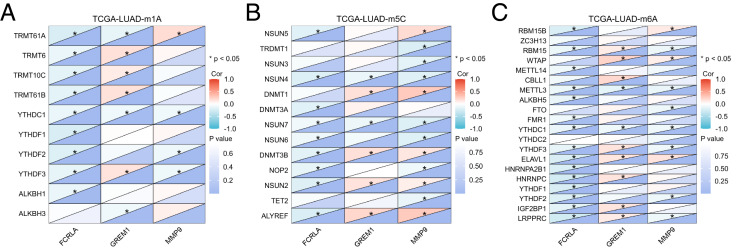
Correlation analysis of target genes and regulators of methylation modification. (A) m1A; (B) m5C; (C) m6A. Correlations depends on the rho value of Pearson and statistical significance (*P < 0.05).

In addition, mRNA methylation modification, as an important component of epigenetics, plays a crucial role in post transcriptional gene regulation. This study focuses on analyzing three common types of mRNA modifications: m1A (N1 methyladenine), m5C (5-methylcytosine), and m6A (N6 methyladenine). The results showed that the expression levels of *FCRLA*, *GREM1*, and *MMP9* were significantly correlated with most m1A, m5C, and m6A modifications in LUAD tissues (Figs. 11A–11C, *P* < 0.05). These results suggest that the expression of target genes may be jointly regulated by DNA methylation and mRNA modification, further supporting their important role in the pathogenesis of LUAD (Fig. 11, Table S1).

### Construction of the lncRNA-miRNA-mRNA regulatory network

**Figure 12:**
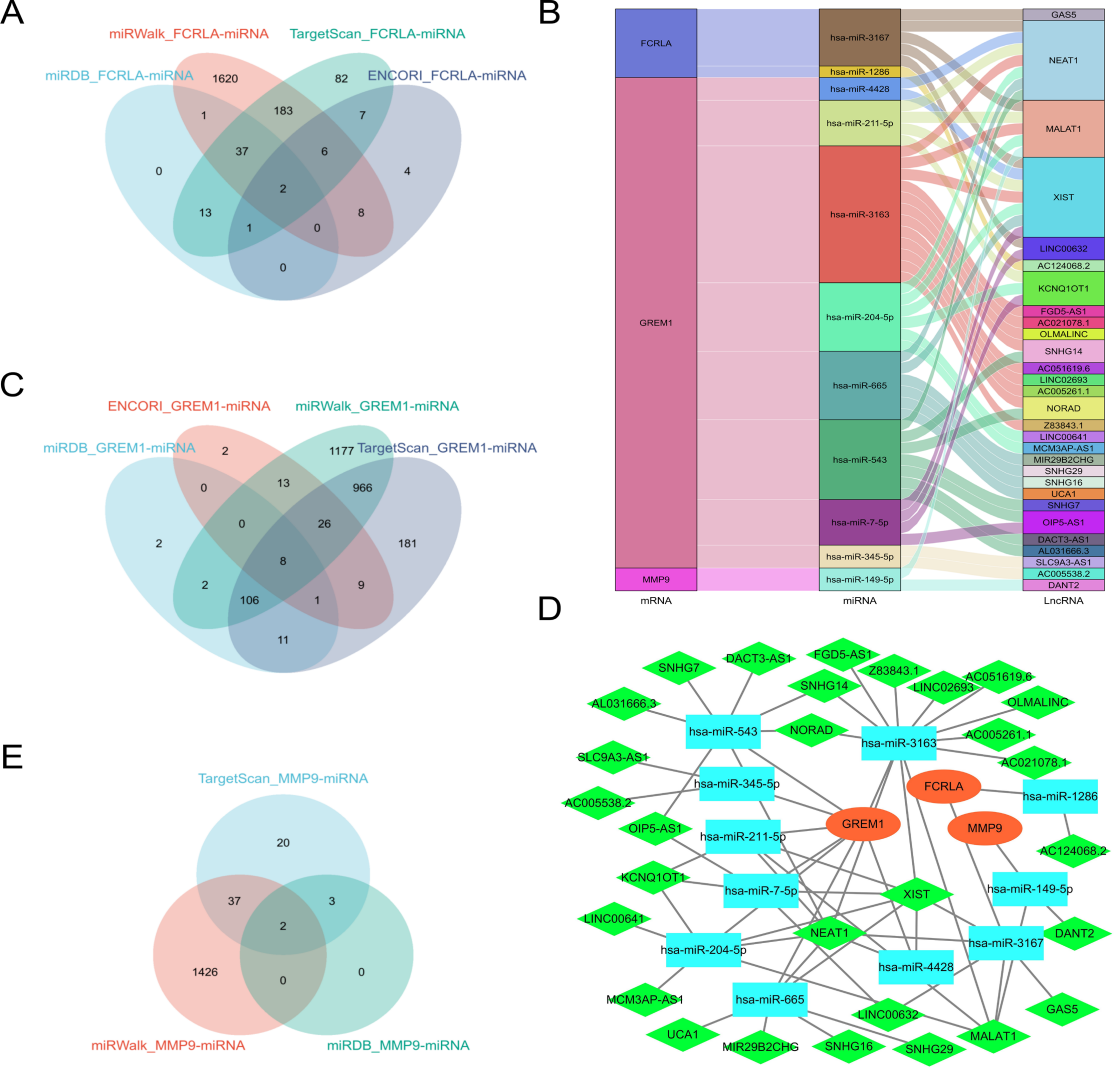
Key ceRNA network construction related to the target genes. (A, C, E) Venn diagram of the predicted *FCRLA*, *GREM1*, and *MMP9* target miRNA in miRWalk, miRDB, ENCORI, and Targetscan. (B) A Sankey plot of the target miRNA and its target lncRNA. (D) lncRNA-miRNA-mRNA regulatory network constructed using Cytoscape, with orange oval for mRNA, blue square for miRNA, and green diamond for LncRNA.

Predict target miRNAs for *FCRLA, GREM1*, and *MMP9* using four databases: miRWalk (Sticht et al., 2018), miRDB (Chen & Wang, 2020), ENCORI (Cui et al., 2024), and TargetScan (Mon-López & Tejero-González, 2019). Define miRNAs that are cross-validated in at least three of these databases as target miRNAs. The analysis results showed that *FCRLA* has two target miRNAs, *GREM1* has eight target miRNAs, and *MMP9* has two target miRNAs (Figs. 12A, 12C, 12E). These miRNAs may regulate the expression of target genes at the post transcriptional level, thereby participating in the occurrence and development of LUAD. Subsequently, an lncRNA miRNA interaction network was constructed using the StarBase2.0 (Li et al., 2014) database, and combined with mRNA, the regulatory relationship of lncRNA miRNA mRNA was further analyzed. The generated Sankey plot (Fig. 12B) and PPI plot (Fig. 12D) demonstrate the complex associations between key lncRNAs, miRNAs, and target genes. This network reveals multi-level gene regulation patterns, suggesting that *FCRLA*, *GREM1*, and *MMP9* may be co-regulated by multiple non-coding RNAs in the pathological process of LUAD, further enriching the understanding of their functional mechanisms.

### Drug sensitivity analysis of the target genes

**Figure 13:**
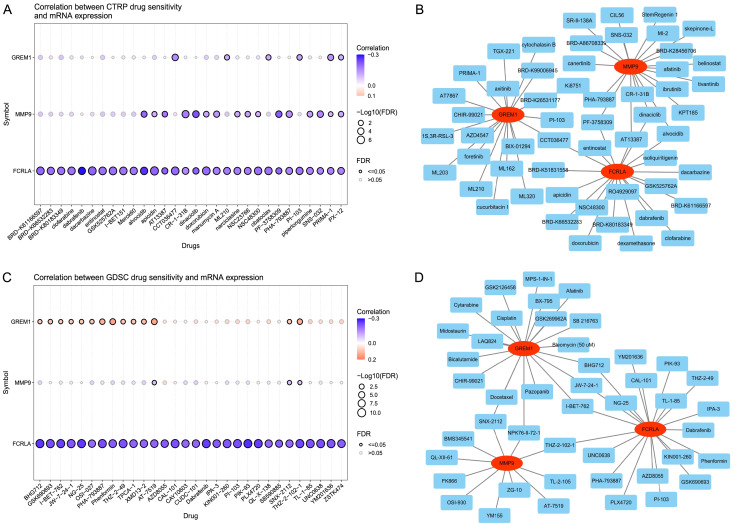
Drug sensitivity of the related drugs. (A) Association between the CTRP drug sensitivity and the expression of *FCRLA*, *GREM1*, and *MMP9*. (B) Drug sensitivity derived from CTRP. (C) Correlation between GDSC drug sensitivity and the expression of *FCRLA*, *GREM1*, and *MMP9*. (D) From the GDSC drug sensitivity.

The analysis results of the CTRP database (Wu et al., 2024) showed that the expression levels of *FCRLA*, *GREM1*, and *MMP9* were significantly correlated with the sensitivity of multiple drugs (Fig. 13A). Drug–target interaction analysis showed that five drugs, CCT036477, PF-3758309, AT13387, dinaciclib, and alvocidib, can simultaneously target at least two of the genes (Fig. 13B), suggesting their potential to jointly regulate pathological mechanisms related to COPD and LUAD. In addition, according to drug sensitivity analysis in the GDSC database, the pharmacological effects of AT-7519, SNX-2112, and THZ-2-102-1 were significantly correlated with the expression of target genes (*P* < 0.05) (Fig. 13C). Further analysis confirmed that drugs such as SNX-2112, NPK 76-II-72-1, THZ-2-102-1, I-BET-762, JW-7-24-1, and BHG712 can simultaneously act on two or more target genes (Fig. 13D). These results indicate that these drugs may have potential value in treating COPD and LUAD, providing new directions for personalized treatment.

### Verify the relationship between target genes and COPD by single cell and qPCR

**Figure 14:**
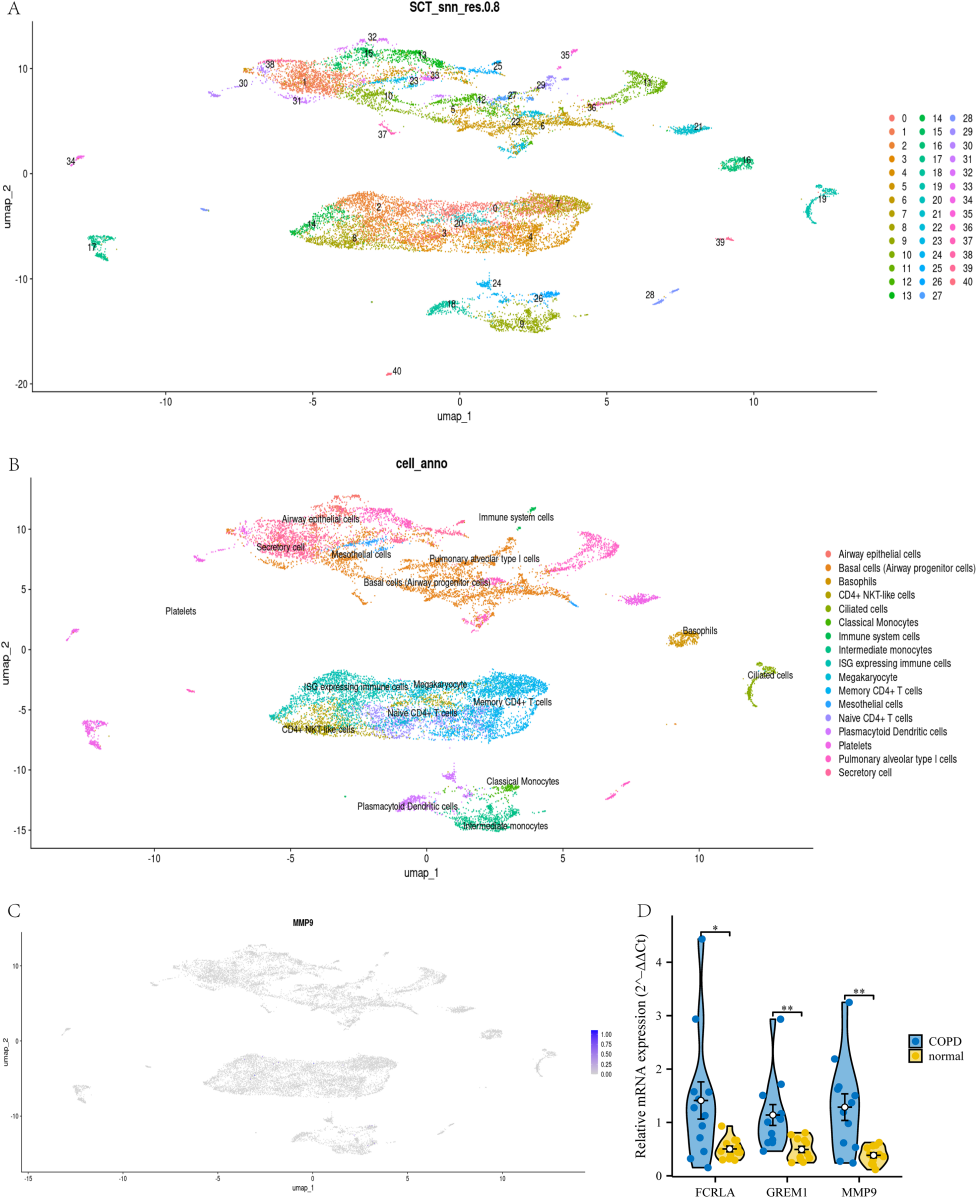
Joint validation of single-cell and qPCR in COPD. (A) UMAP clustering map of GSE270667 dataset; (B) UMAP clustering cell annotation map of GSE270667 dataset; (C) Localization of *MMP9* in UMAP clustering map; (D) QPCR validation results of three key genes. Data are presented as mean ± SD. Statistical significance was determined by two-tailed Student’s t-test (*P < 0.05, **P < 0.01).

To confirm the cell type-specific expression patterns identified in our transcriptome analysis, we analyzed single-cell RNA-seq data. This study used UMAP analysis to divide all cells in the GSE270667 dataset into 40 clusters (see Fig. 14A). These 40 clusters are further subdivided into 17 different cell types, such as CD4+NKT like cells, monocytes, memory CD4+T cells, basophils, mesothelial cells, and immune cells expressing interferon stimulated genes (ISG) (see Fig. 14B). Among these cell types, *MMP9* is mainly highly expressed in airway monocytes and immune cells expressing ISG, while the cell types of the other two genes have not been determined (see Fig. 14C). This fully demonstrates that *MMP9* significantly affects the immune microenvironment of chronic obstructive pulmonary disease (COPD) through high expression in immune cells. To strengthen our findings, we performed qPCR to measure the expression levels of these three target genes in COPD patients and normal controls. The experimental results showed that the expression levels of *FCRLA*, *GREM1*, and *MMP9* were significantly increased in COPD patients, consistent with the validation results of GSE76925 data (see Fig. 14D). These findings not only confirm the important roles of three target genes in the pathological mechanism of COPD, but also suggest that *MMP9* may play a more critical role in specific molecular pathways of COPD.

### Verify the relationship between target genes and LUAD by single cell and qPCR

**Figure 15:**
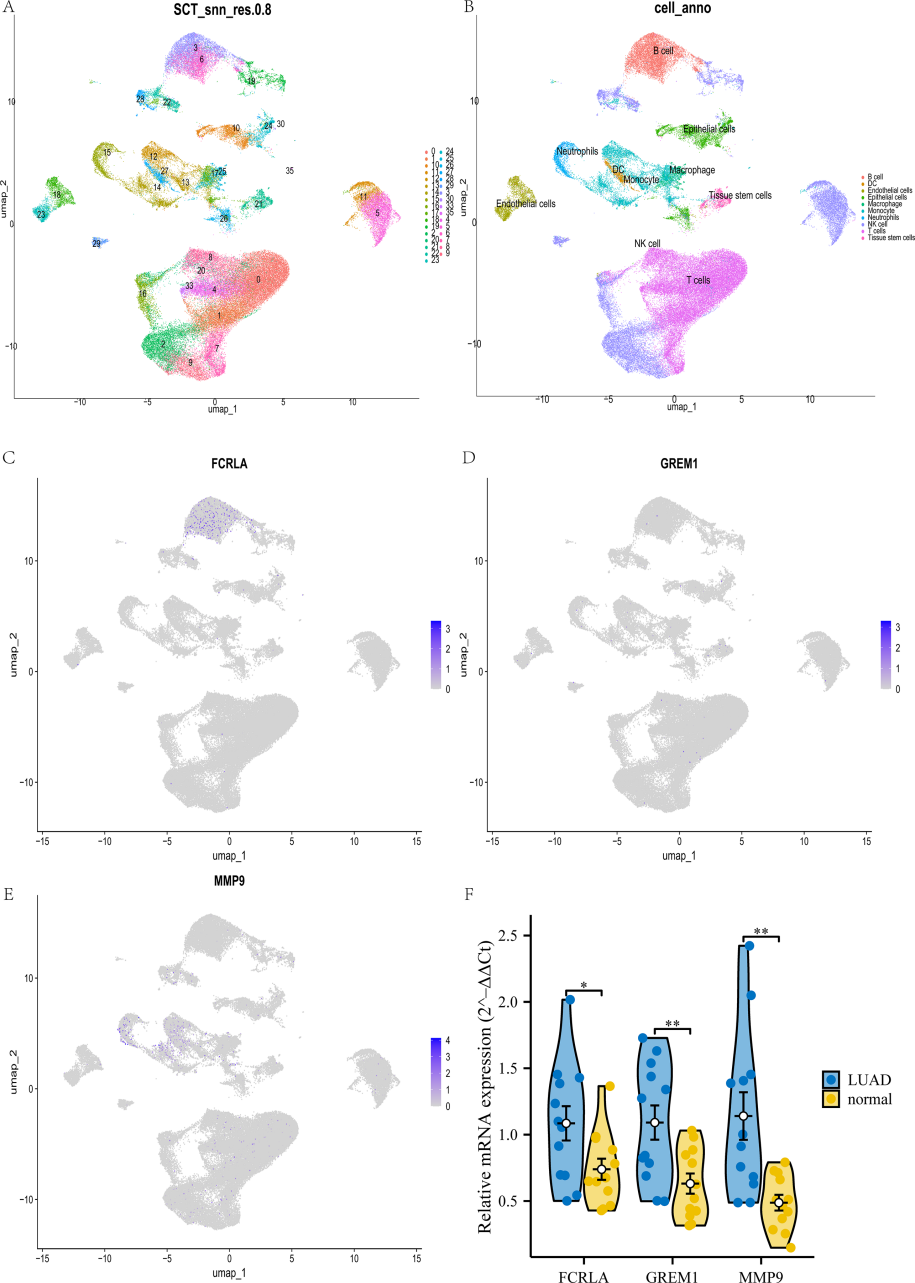
Joint validation of single-cell and qPCR in LUAD. (A) UMAP clustering map of GSE189357 dataset; (B) UMAP clustering cell annotation map of GSE189357 dataset; (C–E) localization of *FCRLA*, *GREM1*, and *MMP9* in UMAP clustering map; (F) QPCR validation results of three key genes. Data are presented as mean ± SD. Statistical significance was determined by two-tailed Student’s t-test (*P < 0.05, **P < 0.01).

This study used UMAP analysis to divide all cells in the GSE189357 dataset into 35 groups (see Fig. 15A). These 35 clusters are further subdivided into 10 different cell types, including NK like cells, monocytes, T cells, B cells, macrophages, *etc*. (see Fig. 15B). Among these cell types, *FCRLA* is mainly expressed in B cells (see Fig. 15C), *GREM1* is mainly expressed in T cells (see Fig. 15D), and *MMP9* is mainly highly expressed in monocytes (see Fig. 15E), which is consistent with the previous finding of high expression of *MMP9* in COPD monocytes. In addition, to enhance the rigor of our research, we further validated the expression levels of these three target genes in lung adenocarcinoma (LUAD) patients and normal controls through qPCR experiments. The experimental results showed that the expression levels of *FCRLA*, *GREM1*, and *MMP9* were significantly increased in LUAD, which was consistent with the validation results of TCGA-LUAD data (see Fig. 15F). These results demonstrate the important roles of *FCRLA* and *GREM1* in the pathology of LUAD. They also suggest that *MMP9*, acting through monocytes, may have a critical role in the molecular pathways of both COPD and LUAD.

## DISCUSSION

Chronic obstructive pulmonary disease (COPD) and lung adenocarcinoma (LUAD) are common respiratory diseases with high incidence and mortality rates (Li & Liu, 2024). Clinical and epidemiological studies have shown that COPD is an independent risk factor for LUAD; however, the exact molecular mechanisms linking the two remain unclear (Shakeel et al., 2023; Hoang & Landi, 2022). This study adopts a multi-omics approach, combining multidimensional strategies such as Mendelian randomization (MR) (Liao et al., 2024), differential expression analysis, immune infiltration, epigenetics, drug target prediction, and single-cell transcriptome sequencing. We identified three key genes—*FCRLA, GREM1*, and *MMP9*—and thoroughly explored their cell-specific expression patterns and functional roles in COPD and LUAD, providing a new theoretical basis to uncover their comorbidity mechanisms.

Through differential expression analysis, we identified 31 common differentially expressed genes from the COPD (GSE76925) (Ashique et al., 2025) and LUAD (GSE116959) (Myszko et al., 2025) datasets. These genes were enriched in pathways closely related to chronic inflammation and the tumor microenvironment, including extracellular matrix remodeling, inflammatory cytokine regulation, and the PI3K-Akt pathway. Subsequently, through LASSO regression screening, we further focused on three key genes that were significantly upregulated in both diseases: *FCRLA*, *GREM1*, and *MMP9*, and constructed a risk scoring model based on these three genes. These genes may represent shared molecular features between COPD and LUAD, potentially contributing to the process of tissue remodeling caused by chronic inflammation, which may lead to malignant transformation.

This study’s key innovation lies in the systematic mining and integrated analysis of single-cell transcriptome data from COPD and LUAD samples. Through UMAP dimensionality reduction clustering of the GSE270667 (COPD) and GSE189357 (LUAD) datasets, we identified multiple specific subgroups of immune cells and epithelial cells in COPD and LUAD samples, and clarified the differential expression patterns of three key genes in cell types. *MMP9*, a matrix metalloproteinase, plays a crucial role in extracellular matrix remodeling, tumor invasion, and angiogenesis (Zhou et al., 2025). In COPD samples, it is highly expressed in monocytes and interferon-stimulated gene (ISG)-related immune cells, suggesting a dominant role in inflammation amplification and cell migration. Similarly, in LUAD, MMP9 shows significant expression in monocytes, indicating its key role in the inflammation-tumor continuum. *FCRLA* is mainly expressed in the B cell population of LUAD. As an immunoglobulin-related molecule, its abnormal expression may affect antigen presentation and tumor immune regulation by tumor-associated B cells (Tolnay, 2022). *GREM1* is enriched in the T cell population in LUAD as an antagonist of the BMP signaling pathway, and its regulatory function may involve T cell differentiation and tumor immune tolerance (Davis et al., 2015). These results suggest that the three genes are commonly upregulated in COPD and LUAD but show significant cell specificity, particularly in immune cells. This differential expression may be a key mechanism by which COPD promotes LUAD transformation.

Further ssGSEA analysis (Huang et al., 2021) revealed that the expression levels of *FCRLA*, *GREM1*, and *MMP9* are closely related to various immune cells (such as CD8+T cells, B cells, monocytes, *etc*.), indicating that they may participate in disease progression by regulating the tumor immune microenvironment. This is consistent with the cell localization results in single-cell analysis, indicating that we should not only focus on gene expression changes at the whole tissue level, but also pay attention to dynamic expression and their interactions in specific cell types. This is of great significance for a deeper understanding of the immunopathological processes of COPD and LUAD.

We analyzed DNA methylation and mRNA modification and found that *FCRLA* and *MMP9* showed low methylation levels in LUAD. In contrast, *GREM1* exhibited high methylation. These results suggest that DNA methylation may regulate key gene expression by either upregulating or downregulating them. At the same time, the expression of the three is significantly associated with various mRNA modifications (m1A, m5C, m6A), indicating that post transcriptional modifications are equally important for their regulation. These epigenetic changes may contribute to cell-specific expression regulation mechanisms, thereby mediating functional alterations in immune cells or sustaining activation of inflammatory signaling pathways.

This study also constructed a ceRNA network covering lncRNA, miRNA, and mRNA to further elucidate the non-coding regulatory background of the three major genes. Meanwhile, through the analysis of GDSC and CTRP drug susceptibility databases (Wu et al., 2024), multiple small molecule drugs significantly correlated with key gene expression levels were identified, such as AT-7519, SNX-2112, dinaciclib, *etc*. These drugs may have potential application value in the intervention of COPD to LUAD. It is worth noting that some drugs can act on two or more target genes simultaneously, with the potential for synergistic regulation.

For experimental verification, we validated the significant upregulation of the three key genes in patients’ peripheral blood. This was achieved by qPCR detection of clinically collected blood samples from COPD and LUAD patients. This is highly consistent with the results of TCGA and GEO data analysis, further supporting its feasibility as a stable and detectable potential biomarker.

Although Mendelian randomization, multi-omics, and single-cell fusion analysis were conducted in this study, and the research methods were reasonable and robust, there are still some limitations:

1) Lack of experimental validation: The findings are primarily based on bioinformatics and Mendelian randomization (MR) analyses, which lack direct wet lab validation to confirm the underlying biological mechanisms. Experimental studies are essential to verify these findings and provide more concrete evidence for their biological relevance.
2) The relevance assumption was strongly supported in this study, as all instrumental variables demonstrated F-statistics > 10. However, the independence assumption remains a limitation of MR studies in general, as unmeasured confounders could still influence the results despite sensitivity analyses. Future studies with larger datasets and multi-ethnic populations are required to further validate these findings.
3) Small sample size: The GEO datasets (GSE116959 and GSE76925) had relatively small sample sizes. This limitation may reduce the generalizability and statistical power of the results. In addition, although qPCR validation was supportive, it was conducted in a relatively small cohort (*n* = 12 per group) and measured gene expression in whole blood rather than lung tissue, which may not fully reflect tissue-specific expression patterns. Future studies should include larger cohorts to improve the robustness of these findings across diverse populations.
4) Absence of clinical validation: The identified biomarkers and risk scoring models have not been clinically validated, leaving their real-world utility unverified. Clinical trials and patient cohorts are needed to assess the practical value and diagnostic/prognostic performance of these biomarkers in real-world settings.
5) Batch effects in datasets: Although efforts were made to normalize the data, using multiple datasets may introduce batch effects that could influence the results. Future studies should implement more stringent data harmonization methods to minimize such confounding factors.
6) Population-specific genetic effects: The MR analysis in this study was based on specific populations, and the generalizability of these results to other ethnic groups remains unclear. Expanding the analysis to include diverse populations would enhance the applicability of the findings across different genetic backgrounds.
7) While our integrated multi-omics approach identified promising associations, the predominantly correlative nature of these analyses limits causal inference. Although MR supports a genetic potential relationship between COPD and LUAD, the roles of the specific genes identified require experimental validation. Future studies using *in vitro* and *in vivo* models are needed to establish mechanistic causality.

Future research should focus on experimental validation of these findings in both *in vitro* and *in vivo* models. Expanding cohort sizes, including more diverse populations, and integrating multi-omics data, such as proteomics and metabolomics, would further refine these biomarkers. Longitudinal studies should also be conducted to assess how these biomarkers correlate with disease progression and response to treatment. Ultimately, integrating these findings into clinical trials could lead to the development of personalized therapeutic strategies that improve patient outcomes in COPD and LUAD.

In summary, this study systematically revealed the common molecular mechanisms between COPD and LUAD. Multiple approaches were used, including Mendelian randomization, global transcriptome analysis, immune microenvironment assessment, and single-cell expression profiling. For the first time, the cell-specific roles of *FCRLA*, *GREM1*, and *MMP9* in disease progression were proposed, highlighting the unique advantages of single-cell analysis in revealing key cell populations and pathways involved in disease progression. In the future, by combining spatial transcriptomics, functional validation, and clinical prognostic data, it is expected to promote the clinical translation and application of these biomarkers for early screening and LUAD targeted therapy in high-risk COPD populations.

## Supplemental Information

10.7717/peerj.20672/supp-1 Supplemental Information 1 Lasso analysis.

10.7717/peerj.20672/supp-2 Supplemental Information 2 Codebook for Lasso_analysis.R.

10.7717/peerj.20672/supp-3 Supplemental Information 3 Data.

10.7717/peerj.20672/supp-4 Supplemental Information 4 Code.

10.7717/peerj.20672/supp-5 Supplemental Information 5 STROBE checklist.

10.7717/peerj.20672/supp-6 Supplemental Information 6 MIQE checklist.

10.7717/peerj.20672/supp-7 Supplemental Information 7 Supplemental table.

## Additional Information and Declarations

### Microarray Data Deposition

The following information was supplied regarding the deposition of microarray data:

The TCGA-LUAD data is available at Zenodo: LI,. Yong. (2024). Machine learning and bioinformatics analysis of diagnostic biomarkers associated with the occurrence and development of lung adenocarcinoma [Data set]. Zenodo. https://doi.org/10.5281/zenodo.12561085.

The raw sequence data are available at NCBI GEO database: GSE76925, GSE116959, GSE270667, GSE189357.

### Data Availability

The following information was supplied regarding data availability:

The data and code are available in the Supplemental Files.

The TCGA-LUAD data is available at Zenodo: LI,. Yong. (2024). Machine learning and bioinformatics analysis of diagnostic biomarkers associated with the occurrence and development of lung adenocarcinoma [Data set]. Zenodo. https://doi.org/10.5281/zenodo.12561085.

The raw sequence data are available at NCBI GEO database: GSE76925, GSE116959, GSE270667, GSE189357.
