# Supplementary material for: Single cell atlas of the comorbidity mechanism between chronic obstructive pulmonary disease and lung adenocarcinoma: a study of multi-omics combined analysis
Source: PeerJ. 2026 Feb 6;14:e20672. doi: 10.7717/peerj.20672 (PMC12884963; doi:10.7717/peerj.20672)
Supplement: Supplemental Information 2 [file peerj-14-20672-s002.docx]

#### Chapter 3 Part 4 Optimal Prognostic Model Construction ----

setwd("C:/Users/Administrator/Desktop/Academic1/TCGA Alternative Website Data - ucsc")

### 1 Organize Clinical Data ----

## Download clinical file from official website

##install.packages("tidyverse")

library(tidyverse)

clinical <- read_tsv("GDC_download/clinical.cart.2022-06-21/clinical.tsv")

## Clinical file from UCSC-Xena (already organized, convenient to use, will use as example)

LUAD_surv <- read_tsv("TCGA-LUAD.survival.tsv") %>%

select(-3)

LUAD_phenotype <- read_tsv("TCGA-LUAD.GDC_phenotype.tsv.gz") %>%

column_to_rownames("submitter_id.samples")

### (1) Missing Values - Multiple Imputation ----

## Check for missing values (NA)

source(file = 'Function/FindoutNA.R', encoding = "utf-8")

FindoutNA(LUAD_phenotype)

missing.percent <- unlist(lapply(LUAD_phenotype,

function(x)sum(is.na(x))))/nrow(LUAD_phenotype) # Calculate percentage of missing per column

## Multiple Imputation (MI)

## 1) Remove variables with more than 20% missing values ----

LUAD_phenotype <- LUAD_phenotype[,missing.percent<=0.2]

col_name <- colnames(LUAD_phenotype) %>% as.data.frame()

LUAD_phenotype <- LUAD_phenotype %>%

dplyr::select(1,21,13,14,15,32,33) %>%

set_names("age","smoking_history","M",

"N","T","gender","race") %>%

rownames_to_column("sample")

## Outlier issue

summary(LUAD_phenotype$age)

summary(LUAD_phenotype$smoking_history)

## albumin creatinine `platelet count` have outliers

write_tsv(LUAD_phenotype,file = "LUAD_phenotype.tsv")

## Delete outlier rows in Excel - albumin creatinine `platelet count' bmi

LUAD_phenotype1 <- read_tsv("LUAD_phenotype.tsv") %>%

column_to_rownames("sample")

## 2) Visualize missingness ----

## ①First column shows number of missing, last column shows number of factors missing ##

## install.packages('mice')

library(mice)

md.pattern(LUAD_phenotype1)

## ②Explore characteristics of missing values with graphs ###

## install.packages("VIM")

library(VIM)

## Left plot shows count of missing (bar chart); right shows proportion missing per factor

aggr(LUAD_phenotype1,

numbers = T, ## Whether to display numbers

prop=T) ## Show proportion? or count?

## ③Visualize missing vs. present per sample ##

## install.packages("Amelia")

library(Amelia)

missmap(LUAD_phenotype1, col = c("white", "lightblue"))

## 3) Multiple Imputation ----

LUAD_phenotype2 <- LUAD_phenotype1 %>% dplyr::select(1,2,3)

library(ggplot2)

library(ggpubr)

## Data are not independent of each other ##

imp <- mice(LUAD_phenotype2,

m=5, ## Number of imputed datasets, 5 is conventional

seed=1234, ## Random seed

method = "cart") ## Imputation algorithm

LUAD_phenotype3 <- mice::complete(imp)

LUAD_phenotype1 <- LUAD_phenotype1 %>%

dplyr::select(-c(2,3,1)) %>%

rownames_to_column("sample")

LUAD_phenotype4 <- LUAD_phenotype3 %>% rownames_to_column("sample")

LUAD_phe <- merge(LUAD_phenotype1,LUAD_phenotype4,by = "sample") %>%

na.omit()

## 4) Validate imputed data ----

## Imputed data distribution similar to original (mean sd p>0.05)

source(file = 'Function/GetTable_comparet.R',

encoding = "utf-8")

GetTable_compare(data1 = LUAD_phenotype2,

data2 = LUAD_phenotype3,

df_name = 'Validation after imputation')

write_tsv(LUAD_phe,file = "LUAD_phe_final.tsv")

## (2) Data format conversion ----

## Delete 'not reported' in tumor_stage in Excel

LUAD_phe <- read_tsv("LUAD_phe_final.tsv") %>%

column_to_rownames("sample")

str(LUAD_phe)

table(LUAD_phe$age)

table(LUAD_phe$M)

table(LUAD_phe$N)

table(LUAD_phe$T)

table(LUAD_phe$gender)

table(LUAD_phe$race)

## character to factor

LUAD_phe$age <- ifelse(LUAD_phe$age<60,

"<60",

">=60")

LUAD_phe$T <- ifelse(LUAD_phe$T == "T1"|LUAD_phe$T == "T2",

"T1_2",

"T3_4") # | means 'or'

LUAD_phe$race <- ifelse(LUAD_phe$race == "asian",

"asian",

"others")

LUAD_phe$age <- factor(LUAD_phe$age)

LUAD_phe$gender <- factor(LUAD_phe$gender)

LUAD_phe$M <- factor(LUAD_phe$M)

LUAD_phe$N <- factor(LUAD_phe$N)

LUAD_phe$T <- factor(LUAD_phe$T)

LUAD_phe$smoking_history <- factor(LUAD_phe$smoking_history)

LUAD_phe$race <- factor(LUAD_phe$race)

str(LUAD_phe)

LUAD_phe1 <- LUAD_phe %>% rownames_to_column("sample")

## (3) Baseline characteristics table ----

source('Function/GetTable1.R', encoding = "utf-8")

GetTable1(df = LUAD_phe, # df input data frame to organize

df_name = 'baseline') # df_name name of output table

### 2 Univariate COX Analysis ----

library(rms) ## Load R package

library(ResourceSelection)

library(dplyr)

library(caret)

library(plyr)

library(survival)

library(readr)

hubgene <- read_csv("TCGA-LUAD.htseq_counts.tsv/lasso regression special table.csv")

## (1) Data organization: 15 differential gene expression matrix and corresponding clinical data ----

## Organize survival data (days to months)

LUAD_surv1 <- LUAD_surv %>%

mutate(OS_time=OS.time/30) %>%

select(-3)

LUAD_surv1$OS_time <- round(LUAD_surv1$OS_time,0)

LUAD_surv2 <- LUAD_surv1 %>% dplyr::filter(OS_time>=1)

## Sample intersection

intersect_sample <- intersect(intersect(hubgene$sample,LUAD_phe1$sample),

LUAD_surv2$sample)

hubgene_1 <- hubgene %>%

as.data.frame() %>%

dplyr::filter(sample%in%intersect_sample)

LUAD_phe2 <- LUAD_phe1 %>%

dplyr::filter(sample%in%intersect_sample)

LUAD_surv3 <- LUAD_surv2 %>% dplyr::filter(sample%in%intersect_sample)

aero_merge_data <- merge(merge(LUAD_surv3,LUAD_phe2,by="sample"),

hubgene_1,by="sample")

save(hubgene_1,LUAD_phe2,LUAD_surv3,aero_merge_data,

file = "hubgene.rda")

write.table(aero_merge_data,"aero_merge_data.csv",row.names=FALSE,col.names=TRUE,sep=",")

write.table(hubgene_1,"hubgene_1.csv",row.names=FALSE,col.names=TRUE,sep=",")

## (2) Univariate COX regression analysis ----

load("hubgene.rda")

BaSurv <- Surv(time = aero_merge_data$OS_time, ## Survival time

aero_merge_data$OS==1) ## Survival status

UniCox <- function(x){ ## Build an R function for later use

FML <- as.formula(paste0('BaSurv~',x)) ## Build survival analysis formula

GCox <- coxph(FML, data = aero_merge_data) ## Cox analysis

GSum <- summary(GCox) ## Output results

HR <- round(GSum$coefficients[,2],2) ## Output HR value

PValue <- round(GSum$coefficients[,5],3) ## Output P value

CI <- paste0(round(GSum$conf.int[,3:4],2),collapse = "-") ## Output HR confidence interval

Unicox <- data.frame("characteristics" = x, ## Return results, build data frame

"Hazard Ratio" = HR,

"CI95" = CI,

"P Value" = PValue)

return(Unicox)

}

set.seed(1)

UniCox(colnames(aero_merge_data)[32]) ## Test if function has errors?

colnames(aero_merge_data)

VarNames <- colnames(aero_merge_data)[19:ncol(aero_merge_data)]

set.seed(2)

UniVar <- lapply(VarNames,UniCox) ## Batch Cox analysis

UniVar_results <- ldply(UniVar,data.frame) ## Organize results into a data frame

## Select variables with P value <0.2 for inclusion in multivariate cox analysis.

(GetFactors <- UniVar_results$characteristics[which(UniVar_results$P.Value < 0.2)] %>% as.character())

save(UniVar,

UniVar_results,

GetFactors,

file = 'Univar_GetFacotrs.rda')

### 3 Lasso regression to select best prognostic genes ----

load('Univar_GetFacotrs.rda')

library("glmnet") ## Load R package

# Install dplyr package

library("dplyr")

hubgene_1 <- hubgene_1 %>% arrange(sample)

hubgene_1 <- select(hubgene_1,-52)

hubgene_2 <- hubgene_1 %>%

column_to_rownames("sample") %>%

as.matrix() ## According to R package requirements, extract part to be selected and convert to matrix

identical(aero_merge_data$sample,hubgene_1$sample)

set.seed(1)

cvfit = cv.glmnet(hubgene_2,

Surv(aero_merge_data$OS_time,aero_merge_data$OS),

nfold=10,

family = 'cox')

pdf('Lasso1.pdf')

plot(cvfit) ## Plot

dev.off()

fit <- glmnet(hubgene_2, Surv(aero_merge_data$OS_time,aero_merge_data$OS),

family = "cox")

pdf('Lasso2.pdf')

plot(fit, label = TRUE)

dev.off()

coef.min = coef(cvfit, s = "lambda.min") ## lambda.min & lambda.1se

active.min = which(coef.min != 0 ) ## Find those regression coefficients not penalized to 0

(lasso_geneids <- colnames(hubgene_1)[active.min]) ## Extract gene names

save(cvfit,fit,lasso_geneids,file = 'lasso_geneids.rda')

lasso_sel_geneids <- aero_merge_data[,colnames(aero_merge_data) %in% lasso_geneids]

write.table(lasso_sel_geneids,"lasso_sel_geneids.csv",row.names=F,col.names=TRUE,sep=",")

### 4 Construct risk score model ----

load('lasso_geneids.rda')

### gene signature calculation

Final_genes_select <- lasso_geneids

fml_KM <- as.formula(paste0('BaSurv~',paste0(Final_genes_select,collapse = '+'))) ## Build a score via multivariate Cox regression

MultiCox_KM <- coxph(fml_KM, data = aero_merge_data)

MultiSum_KM <- summary(MultiCox_KM)

index.min <- MultiSum_KM$coefficients[,1]

(index.min <- as.numeric(index.min))

signature <- as.matrix(subset(aero_merge_data,select = Final_genes_select)) %*%

as.matrix(exp(index.min))

aero_merge_data$signature <- signature

### 5 KM validation ----

library(ggplot2)

library(survminer)

library(survival)

aero_merge_data$signature_2 <- ifelse(aero_merge_data$signature >

median(aero_merge_data$signature),

'High-Score','Low-Score') ## Note naming here!!!

KM_fit <- survfit(Surv(OS_time, OS)~signature_2,data = aero_merge_data)

plot_KM <- ggsurvplot(KM_fit, conf.int=F, pval=T, risk.table=T,

legend.labs = c('High-Score','Low-Score'),

legend.title='Risk Score',

palette = c("dodgerblue2","orchid2"),

risk.table.height = 0.3)

plot_KM

pdf('Signature_KMplot.pdf',onefile = F,width = 8,height = 6)

print(plot_KM)

dev.off()

save(cvfit,fit,KM_fit,plot_KM,file = 'prognosis_model/rda/Uni_plots.rda')

load( 'prognosis_model/rda/Uni_plots.rda')

### 6 Nomogram ----

library(dplyr)

colnames(aero_merge_data)[35] <- 'Multigene'

aero_merge_data1 <- aero_merge_data[,c(1:18,35)]

str(aero_merge_data1)

aero_merge_data1$Multigene <- factor(aero_merge_data1$Multigene)

aero_merge_data1 <- aero_merge_data1 %>%

column_to_rownames("sample")

for (i in 1:ncol(aero_merge_data1)) {

aero_merge_data1[,i] <- as.numeric(aero_merge_data1[,i])}

BaSurv <- Surv(time = aero_merge_data1$OS_time,event = aero_merge_data1$OS)

## (1) Univariate Cox analysis ----

UniCox_nom <- function(x){

FML <- as.formula(paste0('BaSurv~',x))

GCox <- coxph(FML, data = aero_merge_data1)

GSum <- summary(GCox)

HR <- round(GSum$coefficients[,2],2)

PValue <- round(GSum$coefficients[,5],3)

CI <- paste0(round(GSum$conf.int[,3:4],2),collapse = "-")

Unicox <- data.frame("characteristics" = x,

"Hazard Ratio" = HR,

"CI95" = CI,

"P Value" = PValue)

return(Unicox)

}

UniCox_nom(colnames(aero_merge_data1)[5])

VarNames_nom <- colnames(aero_merge_data1)[3:18] ## Select variables to include in univariate Cox regression

set.seed(1)

UniVar_nom <- lapply(VarNames_nom,UniCox_nom) ## Batch univariate Cox analysis

UniVar_nom <- ldply(UniVar_nom,data.frame) ## Organize results into data frame

## From univariate Cox results, select variables with P value <0.05 for inclusion in multivariate Cox analysis.

GetFactors_uni_nom <- UniVar_nom$characteristics[which(UniVar_nom$P.Value < 0.05)] %>% as.character()

write_tsv(UniVar_nom,'prognosis_model/table/UniVar_nom.tsv')

## (2) Multivariate analysis ----

## To exclude multicollinearity issues, need to exclude T, N, M

GetFactors_uni_nom1 <- c("rela_fami_cancer_histo", "race",

"tumor_stage" ,"platelet_count","Multigene" )

fml_nom <- as.formula(paste0('BaSurv~',

paste0(GetFactors_uni_nom1,collapse = '+')))

MultiCox_nom <- coxph(fml_nom, data = aero_merge_data1)

MultiSum_nom <- summary(MultiCox_nom)

MultiName_nom <- as.character(GetFactors_uni_nom1)

MHR_nom <- round(MultiSum_nom$coefficients[,2],2)

MPV_nom <- round(MultiSum_nom$coefficients[,5],3)

MCIL_nom <- round(MultiSum_nom$conf.int[,3],2)

MCIU_nom <- round(MultiSum_nom$conf.int[,4],2)

MCI_nom <- paste0(MCIL_nom,'-',MCIU_nom)

MulCox_nom <- data.frame('characteristics' = MultiName_nom,

'Hazard Ratio' = MHR_nom,

'CI95' = MCI_nom,

'P Value' = MPV_nom)

MulCox_nom1 <- MulCox_nom %>%

as.data.frame() %>%

rownames_to_column("Mulcox") %>%

select(-1)

## Merge univariate and multivariate COX regression results in Excel ##

write_tsv(MulCox_nom1,'prognosis_model/table/MulCox_nom.tsv')

Final <- read_tsv("prognosis_model/table/merge_Mul_COX_results.tsv")

GetFactors_model <- Final$characteristics[which(Final$P.Value.y < 0.05)] ## Determine factors to include in prediction model (multivariate Cox results, P value less than 0.05)

save(GetFactors_model,file = 'prognosis_model/rda/GetFactors_model.rda')

load( 'prognosis_model/rda/GetFactors_model.rda')

## (3) Nomogram ----

str(aero_merge_data1)

aero_merge_data1$Multigene <- factor(aero_merge_data1$Multigene)

aero_merge_data1$age <- factor(aero_merge_data1$age)

aero_merge_data1$histologic_grade <- factor(aero_merge_data1$histologic_grade)

aero_merge_data1$T <- factor(aero_merge_data1$T)

aero_merge_data1$N <- factor(aero_merge_data1$N)

aero_merge_data1$M <- factor(aero_merge_data1$M)

aero_merge_data1$rela_fami_cancer_histo <- factor(aero_merge_data1$rela_fami_cancer_histo)

aero_merge_data1$vascular_tumor_cell_type <- factor(aero_merge_data1$vascular_tumor_cell_type)

aero_merge_data1$gender <- factor(aero_merge_data1$gender)

aero_merge_data1$race <- factor(aero_merge_data1$race)

aero_merge_data1$tumor_stage <- factor(aero_merge_data1$tumor_stage)

dd <- datadist(aero_merge_data1) ## Package data

options(datadist="dd") ## Set global variable

## Check data type

str(aero_merge_data1)

BaSurv.norm <- Surv(time = aero_merge_data1$OS_time,event = aero_merge_data1$OS)

fml.nom <- as.formula(paste0('BaSurv.norm~',paste0(GetFactors_model,collapse = '+'))) ## Build multivariate Cox formula

f <- cph(fml.nom, x=T, y=T, surv=T, data=aero_merge_data1)

surv <- Survival(f)

nom <- nomogram(f, ## Survival function

fun=list(function(x) surv(12, x), ## We want to predict 1-year, 3-year, and 5-year survival rates

function(x) surv(12*3, x),

function(x) surv(12*5, x)),

lp=F, funlabel=c("1-year survival","3-year survival", "5-year survival"), ## label annotation

maxscale=100, ## Maximum score 100

fun.at=seq(0.1,0.9,0.1)) ## Set scale

## Nomogram visualization ##

pdf(paste0('normgram.pdf'),width = 13.5,height = 9, onefile=FALSE)

plot(nom,cex.var = 2,cex.axis = 1.5,lwd = 10,xfrac = 0.5,tcl = 0.5)

dev.off()

save(nom,file = 'prognosis_model/rda/nom.rda')

load('prognosis_model/rda/nom.rda')

### 7 Forest plot ----

Final <- read_tsv("prognosis_model/table/merge_Mul_COX_results.tsv")

## Draw with forestploter package

library(grid)

## install.packages("forestploter")

library(forestploter)

# UniVar_forest

UniVar_forest <- read_tsv("prognosis_model/table/UniVar_nom_forest.tsv")

UniVar_forest$`Forestplot ` <- paste(rep(" ", 20), collapse = " ")

UniVar_forest <- UniVar_forest %>%

relocate(`P.Value`,.after = `Forestplot `)

UniVar_forest$se <- (log(UniVar_forest$CI_high) - log(UniVar_forest$HR))/1.96

tm <- forest_theme(base_size = 10,

refline_col = "red",

footnote_col = "#636363",

footnote_fontface = "italic")

UniVar_p <- forest(

UniVar_forest[,c(1:2,6:7)],

est = UniVar_forest$HR,

lower = UniVar_forest$CI_low,

upper = UniVar_forest$CI_high,

sizes = UniVar_forest$se,

ci_column = 3,

ref_line = 1,

xlim = c(0, 5),

ticks_at = c(0.5, 1, 2, 3,4),

theme = tm)

# Print plot

plot(UniVar_p)

# MulCox_forest

MulCox_forest <- read_tsv("prognosis_model/table/MulCox_nom_forest.tsv")

MulCox_forest$`Forestplot ` <- paste(rep(" ", 20), collapse = " ")

MulCox_forest <- MulCox_forest %>%

relocate(`P.Value`,.after = `Forestplot `)

MulCox_forest$se <- (log(MulCox_forest$CI_high) - log(MulCox_forest$HR))/1.96

tm <- forest_theme(base_size = 10,

refline_col = "red",

footnote_col = "#636363",

footnote_fontface = "italic")

MulCox_p <- forest(

MulCox_forest[,c(1:2,6:7)],

est = MulCox_forest$HR,

lower = MulCox_forest$CI_low,

upper = MulCox_forest$CI_high,

sizes = MulCox_forest$se,

ci_column = 3,

ref_line = 1,

xlim = c(0, 5),

ticks_at = c(0.5, 1, 2, 3,4),

theme = tm)

# Print plot

plot(MulCox_p)

### 8 Time-ROC curve to validate risk model sensitivity and specificity ----

library(timeROC)

cox.timp2 <- coxph(Surv(aero_merge_data1$OS_time,

aero_merge_data1$OS == 1) ~

aero_merge_data1$Multigene)

lpFit <- cox.timp2$linear.predictors

roc.fit <- timeROC(T = aero_merge_data1$OS_time, # Outcome time

delta = aero_merge_data1$OS, # Survival outcome

marker = lpFit, # Predictor

cause = 1, # Positive outcome assignment, e.g., death, recurrence assignment

weighting = "marginal", ## 'marginal' = Kaplan-Meier estimator of the censoring distribution

## or 'cox' = censoring by the Cox model

times = c(12*c(1,3,5,7,10)), ## Time points, select 1-year, 3-year, and 5-year survival rates

ROC = T,

iid = TRUE)

CI95 <- confint(roc.fit)[1] %>% unlist()

ROC_1 <- paste0(' : ',roc.fit$AUC[1] %>% as.numeric() %>% round(.,2),'(',CI95[1],'-',CI95[length(CI95)/2 + 1],')')

ROC_3 <- paste0(' : ',roc.fit$AUC[2] %>% as.numeric() %>% round(.,2),'(',CI95[2],'-',CI95[length(CI95)/2 + 2],')')

ROC_5 <- paste0(' : ',roc.fit$AUC[3] %>% as.numeric() %>% round(.,2),'(',CI95[3],'-',CI95[length(CI95)/2 + 3],')')

ROC_7 <- paste0(' : ',roc.fit$AUC[4] %>% as.numeric() %>% round(.,2),'(',CI95[4],'-',CI95[length(CI95)/2 + 4],')')

ROC_10 <- paste0(' : ',roc.fit$AUC[5] %>% as.numeric() %>% round(.,2),'(',CI95[5],'-',CI95[length(CI95)/2 + 5],')')

col_all <- rainbow(10)

filename <- paste0(paste0('OS_AUC_t_group_TypeI.pdf'))

pdf(filename,onefile = F)

plot(roc.fit,time=12,col = col_all[1],add =FALSE,title = F) #time is time point, col is line color, add means whether to add to previous graph

plot(roc.fit,time=12*3,col = col_all[2],add = TRUE)

plot(roc.fit,time=12*5,col = col_all[3],add = TRUE)

plot(roc.fit,time=12*7,col = col_all[4],add = TRUE)

plot(roc.fit,time=12*10,col = col_all[5],add = TRUE)

title(main = 'Time-dependent ROC curve')

legend("bottomright",

legend = c(paste0('AUC at 1 year',ROC_1),

paste0('AUC at 3 years',ROC_3),

paste0('AUC at 5 years',ROC_5),

paste0('AUC at 7 years',ROC_7),

paste0('AUC at 10 years',ROC_10)),

lty = c("solid","solid","solid","solid","solid"),

col = col_all[1:5],

bty = 'n')

dev.off()
