# Supplementary material for: Single cell atlas of the comorbidity mechanism between chronic obstructive pulmonary disease and lung adenocarcinoma: a study of multi-omics combined analysis
Source: PeerJ. 2026 Feb 6;14:e20672. doi: 10.7717/peerj.20672 (PMC12884963; doi:10.7717/peerj.20672)
Supplement: Supplemental Information 5 [file peerj-14-20672-s005.docx]

**STROBE-MR checklist of recommended items to address in reports of Mendelian randomization studies**^1^ ^2^

| **Item No.** | **Section** | **Checklist item** | **Page No.** | **Relevant text from manuscript** |
| --- | --- | --- | --- | --- |
| 1 | **TITLE and ABSTRACT** | Indicate Mendelian randomization (MR) as the study’s design in the title and/or the abstract if that is a main purpose of the study | **Page No.:** 1 | **Title: Key Biomarkers and Molecular Mechanisms Linking COPD and LUAD via Mendelian Randomization** |
|  | **INTRODUCTION** |  |  | Abstract: This study aims to investigate the causal relationship between chronic obstructive pulmonary disease (COPD) and lung adenocarcinoma (LUAD) using Mendelian randomization analysis combined with bioinformatics methods. Key biomarkers, including FCRLA, GREM1, and MMP9, were identified and validated, providing insights into shared mechanisms and potential therapeutic targets. |
| 2 | **Background** | Explain the scientific background and rationale for the reported study. What is the exposure? Is a potential causal relationship between exposure and outcome plausible? Justify why MR is a helpful method to address the study question | Page No.: 1 | Chronic obstructive pulmonary disease (COPD) and lung adenocarcinoma (LUAD) are global health challenges with significant morbidity and mortality. COPD increases the risk of LUAD, suggesting shared biological mechanisms. Mendelian randomization (MR) is a valuable tool to infer causal relationships while minimizing confounding factors. This study explores the genetic and molecular basis linking these diseases. |
| 3 | **Objectives** | State specific objectives clearly, including pre-specified causal hypotheses (if any). State that MR is a method that, under specific assumptions, intends to estimate causal effects | Page No.: 1 | The study aims to investigate the causal relationship between COPD and LUAD, identify differentially expressed genes (DEGs) using MR analysis, and determine key biomarkers such as FCRLA, GREM1, and MMP9. These biomarkers were further analyzed for their role in immune regulation and therapeutic relevance. |
|  | **METHODS** |  |  |  |
| 4 | **Study design and data sources** | Present key elements of the study design early in the article. Consider including a table listing sources of data for all phases of the study. For each data source contributing to the analysis, describe the following: | **Page No.:** 2-3 | This study used a two-sample MR framework. COPD (ebi-a-GCST90018807) was the exposure, and LUAD (ebi-a-GCST004744) was the outcome. Gene expression data were sourced from TCGA and GEO databases (datasets GSE116959 and GSE76925). The R (version 4.2.1) software and TwoSampleMR package were utilized for analysis. |
|  | a) | Setting: Describe the study design and the underlying population, if possible. Describe the setting, locations, and relevant dates, including periods of recruitment, exposure, follow-up, and data collection, when available. | Page No.: 2 | This study used a two-sample Mendelian randomization (MR) framework. COPD was defined as the exposure (GWAS ID: ebi-a-GCST90018807) and LUAD as the outcome (GWAS ID: ebi-a-GCST004744). Gene expression data were retrieved from the GEO database (datasets GSE116959 and GSE76925). The study covered LUAD patients, COPD cases, and healthy controls. Data collection spanned across TCGA and GEO, with analyses conducted using R software (version 4.2.1) in 2023. |
|  | b) | Participants: Give the eligibility criteria, and the sources and methods of selection of participants. Report the sample size, and whether any power or sample size calculations were carried out prior to the main analysis | **Page No.:** 5 | Participants were derived from publicly available datasets:  GSE116959 dataset: 57 LUAD patients and 11 healthy controls.  GSE76925 dataset: 111 COPD cases and 40 smoking controls.  Inclusion criteria were defined by the original studies in GEO. The total sample size was sufficient for identifying significant differentially expressed genes (DEGs) and conducting Mendelian randomization analyses. |
|  | c) | Describe measurement, quality control and selection of genetic variants | **Page No.:** 3 | Genetic variants for COPD were extracted from the GWAS dataset (ebi-a-GCST90018807) using a threshold of p < 5 × 10⁻⁸ for genome-wide significance. Quality control measures ensured that variants met Hardy-Weinberg equilibrium and linkage disequilibrium criteria. Instrumental variables were selected based on their association with COPD and independence from confounding factors. The GWAS dataset for LUAD (ebi-a-GCST004744) was used as the outcome dataset. |
|  | d) | For each exposure, outcome, and other relevant variables, describe methods of assessment and diagnostic criteria for diseases | **Page No.:** 2 | COPD and LUAD were assessed using clinical and genetic data from GWAS and GEO datasets. Diagnostic criteria for COPD were based on spirometry measurements (FEV1/FVC < 0.7), while LUAD diagnoses were histopathologically confirmed in TCGA and GEO. Gene expression levels in GEO datasets (GSE116959 and GSE76925) were analyzed using R software to identify differentially expressed genes (DEGs). |
|  | e) | Provide details of ethics committee approval and participant informed consent, if relevant | **Page No.:** 9 | The study protocol was approved by the Medical Ethics Committee of Huzhou First People's Hospital (Approval Number: 2023KYLL014). Ethical standards were adhered to, and informed consent was obtained for patient data included in supplementary qPCR validation. |
| 5 | **Assumptions** | Explicitly state the three core IV assumptions for the main analysis (relevance, independence and exclusion restriction) as well assumptions for any additional or sensitivity analysis | **Page No.:** 3 | Three core MR assumptions were met: (1) genetic variants are associated with COPD, (2) variants are not confounded by unmeasured factors, and (3) variants affect LUAD only through COPD. |
| 6 | **Statistical methods: main analysis** | Describe statistical methods and statistics used | Page No.: 5 | Statistical methods included MR analysis using IVW, MR Egger, and MR-PRESSO to estimate causal effects. LASSO regression was applied to identify key biomarkers, and risk models were constructed using R (4.2.1) and ggplot2. Multiple testing corrections were addressed using false discovery rates (FDR). |
|  | a) | Describe how quantitative variables were handled in the analyses (i.e., scale, units, model) | Page No.: 5 | Quantitative variables, such as genetic variant-exposure and variant-outcome associations, were analyzed using beta coefficients and standard errors. For gene expression data, log2-transformed fold change (\|logFC\| ≥ 1) and p-values (p < 0.05) were used to identify differentially expressed genes (DEGs). Risk scoring models were constructed based on normalized gene expression levels. |
|  | b) | Describe how genetic variants were handled in the analyses and, if applicable, how their weights were selected | Page No.: 3-4 | Genetic variants associated with COPD were selected as instrumental variables (IVs) from GWAS datasets using a significance threshold of p < 5 × 10⁻⁸. SNP weights were derived from their effect sizes (beta values) on COPD. LD pruning (r² < 0.01) was performed to ensure independence of the selected SNPs. |
|  | c) | Describe the MR estimator (e.g. two-stage least squares, Wald ratio) and related statistics. Detail the included covariates and, in case of two-sample MR, whether the same covariate set was used for adjustment in the two samples | **Page No.:** 3-4 | The study employed inverse variance weighting (IVW) as the primary MR estimator, supplemented by MR Egger, weighted median, and MR-PRESSO to assess robustness. For two-sample MR, summary statistics for exposure and outcome datasets were harmonized to align alleles. No additional covariates were included in the GWAS summary data, ensuring consistency across both samples. |
|  | d) | Explain how missing data were addressed | **Page No.:** 3-4 | Missing data in the exposure and outcome datasets were addressed through imputation methods within the GWAS summary statistics. Variants with incomplete information on beta coefficients or standard errors were excluded from the analysis. |
|  | e) | If applicable, indicate how multiple testing was addressed | **Page No.:** 3-4 | Multiple testing correction was performed using false discovery rate (FDR) adjustments. For differential gene expression analysis, a threshold of adjusted p < 0.05 was applied. Sensitivity analyses (e.g., leave-one-out and heterogeneity tests) ensured robustness of the findings. |
| 7 | **Assessment of assumptions** | Describe any methods or prior knowledge used to assess the assumptions or justify their validity | **Page No.:** 7 | MR assumptions were assessed using sensitivity analyses such as leave-one-out analysis and heterogeneity tests (e.g., Cochran’s Q). Pleiotropy was evaluated using MR Egger intercept and MR-PRESSO. |
| 8 | **Sensitivity analyses and additional analyses** | Describe any sensitivity analyses or additional analyses performed (e.g. comparison of effect estimates from different approaches, independent replication, bias analytic techniques, validation of instruments, simulations) | **Page No.:** 9 | Sensitivity analyses confirmed the robustness of the findings. Five MR methods (IVW, MR Egger, Weighted Median, Simple Mode, and MR-PRESSO) showed consistent directions and significant associations, supporting the causal relationship between COPD and LUAD. |
| 9 | **Software and pre-registration** |  |  |  |
|  | a) | Name statistical software and package(s), including version and settings used | **Page No.:**3 | Statistical analyses were conducted using R software (version 4.2.1). Key packages included:  TwoSampleMR (version 0.6.0): for Mendelian randomization analyses.  ggplot2 (version 3.3.6): for data visualization (e.g., scatter plots, forest plots).  pROC (version 1.18.0): for ROC curve analysis.  STRING and Cytoscape (version 3.7.2): for protein-protein interaction (PPI) network construction.  All settings followed package defaults unless otherwise specified. |
|  | b) | State whether the study protocol and details were pre-registered (as well as when and where) | **Page No.:** 9 | This study was not pre-registered. The analyses were conducted using publicly available data from GWAS, TCGA, and GEO databases. All methods and results are reproducible with the provided data sources and R scripts. |
|  | **RESULTS** |  |  |  |
| 10 | **Descriptive data** |  | **Page No.:** 4 | The GSE116959 dataset included 57 LUAD samples and 11 healthy controls, while the GSE76925 dataset had 111 COPD cases and 40 smoking controls. Differentially expressed genes (31 DEGs: 15 upregulated, 16 downregulated) were identified. |
|  | a) | Report the numbers of individuals at each stage of included studies and reasons for exclusion. Consider use of a flow diagram | **Page No.:** 5-6 | The GSE116959 dataset included 57 LUAD patients and 11 healthy controls, while the GSE76925 dataset consisted of 111 COPD cases and 40 smoking controls. No individuals were excluded from the analysis as all samples met the inclusion criteria defined by the original studies. A flow diagram illustrating the dataset selection process is provided in Figure 1. |
|  | b) | Report summary statistics for phenotypic exposure(s), outcome(s), and other relevant variables (e.g. means, SDs, proportions) | **Page No.:** 6 | In GSE116959, LUAD samples had significantly higher expression levels of identified biomarkers (e.g., FCRLA, GREM1, and MMP9) compared to healthy controls (mean log2FC ≥ 1, p < 0.05). In GSE76925, COPD samples also showed elevated biomarker expression levels compared to controls. Full summary statistics, including mean and SD values, are available in the supplementary materials. |
|  | c) | If the data sources include meta-analyses of previous studies, provide the assessments of heterogeneity across these studies | **Page No.:** 6 | This study did not involve meta-analyses. However, heterogeneity tests (e.g., Cochran’s Q) were conducted for MR analyses to assess consistency across genetic variants. Results showed no significant heterogeneity (p > 0.05), supporting the robustness of the findings. |
|  | d) | For two-sample MR:  i.  Provide justification of the similarity of the genetic variant-exposure associations between the exposure and outcome samples  ii.  Provide information on the number of individuals who overlap between the exposure and outcome studies | **Page No.:** 3-5 | i. Genetic variants used as instrumental variables were selected from GWAS datasets with no overlap between the exposure (COPD; GWAS ID: ebi-a-GCST90018807) and outcome (LUAD; GWAS ID: ebi-a-GCST004744) populations, minimizing bias. Summary statistics from both datasets were harmonized for allele alignment.  ii. No participant overlap was reported between the GWAS datasets, ensuring independent samples for two-sample MR analysis. |
| 11 | **Main results** |  | **Page No.:**9 | MR analysis confirmed a significant causal relationship between COPD and LUAD. Key biomarkers (FCRLA, GREM1, and MMP9) were identified. Functional enrichment analysis highlighted the PI3K-Akt pathway's role in inflammation and tumor progression. |
|  | a) | Report the associations between genetic variant and exposure, and between genetic variant and outcome, preferably on an interpretable scale | **Page No.:** 9 | The Mendelian randomization (MR) analysis revealed significant associations between genetic variants associated with COPD (e.g., SNPs from the GWAS dataset) and LUAD. The instrumental variables for COPD exposure showed a consistent positive effect on LUAD (P < 0.05). Specifically, genetic variants in the COPD-associated SNPs were associated with increased risk for LUAD, with odds ratios for the genetic variants in the exposure group indicating a strong causal relationship between COPD and LUAD. |
|  | b) | Report MR estimates of the relationship between exposure and outcome, and the measures of uncertainty from the MR analysis, on an interpretable scale, such as odds ratio or relative risk per SD difference | **Page No.:** 9 | The MR estimates indicated that COPD is a significant risk factor for LUAD. The odds ratio (OR) for the genetic variants associated with COPD and LUAD was 1.32 (95% CI: 1.05-1.60), suggesting a moderate increase in LUAD risk per standard deviation increase in COPD exposure. All MR estimates were derived using the inverse variance weighting (IVW) method, and uncertainty measures (e.g., confidence intervals) were included for each estimate. |
|  | c) | If relevant, consider translating estimates of relative risk into absolute risk for a meaningful time period | **Page No.:** 9 | The relative risk of LUAD associated with COPD exposure was translated into an absolute risk using the MR estimates. For a COPD patient with a genetic predisposition, the estimated risk of developing LUAD over a 10-year period was approximately 15% higher compared to non-COPD individuals, considering the identified genetic risk factors. |
|  | d) | Consider plots to visualize results (e.g. forest plot, scatterplot of associations between genetic variants and outcome versus between genetic variants and exposure) | **Page No.:** 9 | A forest plot (Figure 2) was used to visualize the MR results, showing the odds ratios for each genetic variant associated with COPD and LUAD. Additionally, scatterplots were included to display the associations between genetic variants and the outcome (LUAD) as well as between genetic variants and the exposure (COPD), providing a clear graphical representation of the causal relationship. |
| 12 | **Assessment of assumptions** |  |  |  |
|  | a) | Report the assessment of the validity of the assumptions | **Page No.:** 9 | The validity of the core assumptions for Mendelian randomization (MR) was assessed through several methods.  Relevance assumption: Genetic variants strongly associated with COPD (p < 5 × 10⁻⁸) were selected as instrumental variables (IVs).  Independence assumption: To test for independence, we performed sensitivity analyses using MR Egger regression, which showed no evidence of pleiotropy (intercept p > 0.05), indicating that the IVs are not associated with confounding factors.  Exclusion restriction assumption: The relationship between the genetic variants and LUAD was found to be mediated only through COPD, as supported by MR-PRESSO analysis, which indicated no significant violations of this assumption. |
|  | b) | Report any additional statistics (e.g., assessments of heterogeneity across genetic variants, such as *I^2^*, Q statistic or E-value) | **Page No.:** 9 | Heterogeneity across genetic variants was evaluated using Cochran’s Q test and I² statistic. The Q statistic showed no significant heterogeneity (p = 0.47), and the I² value was low (I² = 20%), suggesting that the genetic variants used as IVs provided consistent estimates. Additionally, E-values were calculated to assess the robustness of the MR estimates against potential unmeasured confounders. The results indicated that the estimates were robust, with the E-values suggesting that unmeasured confounding would need to have a substantial effect to alter the conclusions. |
| 13 | **Sensitivity analyses and additional analyses** |  |  |  |
|  | a) | Report any sensitivity analyses to assess the robustness of the main results to violations of the assumptions | **Page No.:** 9 | Sensitivity analyses were performed to evaluate the robustness of the main MR results to potential violations of the assumptions.  Leave-one-out analysis: We performed a leave-one-out analysis, where each genetic variant was sequentially removed from the analysis. The results remained consistent, indicating that no single SNP drove the observed associations.  MR Egger regression: This method was used to detect and correct for pleiotropy. The absence of significant pleiotropy (p > 0.05) suggested that the results were not influenced by unmeasured confounding factors.  MR-PRESSO: MR-PRESSO was applied to detect and correct for outliers, with no outliers found, further supporting the robustness of the results. |
|  | b) | Report results from other sensitivity analyses or additional analyses | **Page No.:** 9 | Additional sensitivity analyses were performed using the weighted median method and simple mode method to further assess the robustness of the causal estimates. These alternative methods confirmed the primary MR findings, with similar estimates of causal effects between COPD and LUAD. Furthermore, the consistency between different MR methods (IVW, MR Egger, weighted median) provides additional evidence of the robustness of the results. |
|  | c) | Report any assessment of direction of causal relationship (e.g., bidirectional MR) | **Page No.:** 9 | A bidirectional Mendelian randomization (MR) analysis was performed to assess the causal relationship between COPD and LUAD in both directions. The results showed that COPD significantly increases the risk of LUAD, but no significant causal effect was found from LUAD to COPD, reinforcing the hypothesis that COPD may contribute to the development of LUAD rather than vice versa. |
|  | d) | When relevant, report and compare with estimates from non-MR analyses | **Page No.:** 10-12 | Non-Mendelian randomization (non-MR) analyses, such as traditional regression models based on observational data, were also conducted. These analyses showed similar associations between COPD and LUAD, suggesting that the MR findings are consistent with traditional statistical approaches. However, MR analysis provides stronger evidence of causality by addressing confounding biases inherent in observational studies. |
|  | e) | Consider additional plots to visualize results (e.g., leave-one-out analyses) | **Page No.:** 11-12 | Additional plots, including leave-one-out analyses and funnel plots, were used to visualize the robustness of the MR results. The leave-one-out plot showed that the causal estimates remained stable even after excluding individual SNPs, indicating no significant influence from specific variants. The funnel plot was symmetric, suggesting no substantial publication bias in the included studies. |
|  | **DISCUSSION** |  |  |  |
| 14 | **Key results** | Summarize key results with reference to study objectives | **Page No.:** 14 | This study identified FCRLA, GREM1, and MMP9 as critical biomarkers linking COPD and LUAD. These findings provide evidence of shared immune regulation mechanisms and potential therapeutic targets. |
| 15 | **Limitations** | Discuss limitations of the study, taking into account the validity of the IV assumptions, other sources of potential bias, and imprecision. Discuss both direction and magnitude of any potential bias and any efforts to address them | **Page No.:**15 | The study is limited by the small sample size, lack of experimental validation, and population-specific genetic effects. Future studies with larger, diverse cohorts and experimental validation are needed. |
| 16 | **Interpretation** |  |  |  |
|  | a) | Meaning: Give a cautious overall interpretation of results in the context of their limitations and in comparison with other studies | **Page No.:** 15 | The results of this study suggest a significant causal relationship between chronic obstructive pulmonary disease (COPD) and lung adenocarcinoma (LUAD), with key biomarkers such as FCRLA, GREM1, and MMP9 identified as potential indicators of this relationship. However, it is important to interpret these findings cautiously due to several limitations, including the reliance on observational data and the potential for residual confounding. Furthermore, the findings are based on genetic instruments from GWAS datasets, which may not fully capture the complexity of COPD and LUAD. Other studies have also suggested a link between COPD and LUAD, but this study is the first to provide genetic evidence supporting a causal role for COPD in the development of LUAD. |
|  | b) | Mechanism: Discuss underlying biological mechanisms that could drive a potential causal relationship between the investigated exposure and the outcome, and whether the gene-environment equivalence assumption is reasonable. Use causal language carefully, clarifying that IV estimates may provide causal effects only under certain assumptions | **Page No.:**15 | The underlying biological mechanisms driving the causal relationship between COPD and LUAD likely involve inflammatory pathways, immune dysregulation, and extracellular matrix remodeling. Upregulated genes such as FCRLA, GREM1, and MMP9 are involved in immune response modulation, tissue remodeling, and tumor progression, which may contribute to the progression from COPD to LUAD. However, the gene-environment equivalence assumption—that genetic variants act only through the exposure (COPD) and not through other pathways—needs to be interpreted with caution. While Mendelian randomization provides evidence of a causal effect, the results are valid only under the assumptions of no pleiotropy, no confounding, and exclusion restriction. Thus, although these findings suggest a causal role for COPD in LUAD development, experimental validation is needed to confirm these mechanisms. |
|  | c) | Clinical relevance: Discuss whether the results have clinical or public policy relevance, and to what extent they inform effect sizes of possible interventions | **Page No.:**14 | The findings of this study have significant clinical and public health implications. The identification of FCRLA, GREM1, and MMP9 as biomarkers could help in the early detection of LUAD in COPD patients, enabling more effective surveillance and personalized treatment strategies. Additionally, understanding the causal relationship between COPD and LUAD opens new avenues for targeted therapies aimed at mitigating the progression from COPD to LUAD. Public health policies could benefit from integrating these findings to improve screening protocols for COPD patients, potentially leading to earlier interventions and better clinical outcomes. The potential for these biomarkers to guide immunotherapy or targeted drug development further enhances their clinical relevance. |
| 17 | **Generalizability** | Discuss the generalizability of the study results (a) to other populations, (b) across other exposure periods/timings, and (c) across other levels of exposure | **Page No.:**15 | The generalizability of the study results is subject to several factors:  To other populations: The results are based on data from publicly available GWAS and GEO datasets, which primarily involve populations of European descent. As such, the generalizability of these findings to other ethnic or geographical populations remains uncertain. Further studies including diverse populations are needed to assess whether the observed causal relationship between COPD and LUAD holds across different genetic backgrounds.  Across other exposure periods/timings: The study focused on genetic variants associated with long-term COPD exposure, as measured by genetic risk scores derived from GWAS. It is not clear whether the same causal relationship would hold for individuals with shorter durations of COPD or for those with early-stage disease. Future research examining COPD at different stages, including early exposures and their potential long-term impact, would help clarify this relationship.  Across other levels of exposure: The study primarily focused on individuals with genetically determined COPD risk. While the findings suggest a causal effect, the generalizability of these results to populations with different levels of exposure (e.g., mild versus severe COPD) remains uncertain. The effect of varying exposure levels on the relationship between COPD and LUAD may differ, and further studies exploring different severities of COPD would enhance the understanding of this relationship. |
|  | **OTHER INFORMATION** |  |  |  |
| 18 | **Funding** | Describe sources of funding and the role of funders in the present study and, if applicable, sources of funding for the databases and original study or studies on which the present study is based | **Page No.:**17 | This study was supported by the National Natural Science Foundation of China (82202649) and Zhejiang Provincial Medical and Health Science and Technology Program (2023KY318). |
| 19 | **Data and data sharing** | Provide the data used to perform all analyses or report where and how the data can be accessed, and reference these sources in the article. Provide the statistical code needed to reproduce the results in the article, or report whether the code is publicly accessible and if so, where | **No.:**17 | The data used in this study are publicly available and can be accessed through the following resources:  GWAS Data: The COPD and LUAD GWAS summary statistics were accessed from the GWAS Catalog (COPD: ebi-a-GCST90018807, LUAD: ebi-a-GCST004744).  Gene Expression Data: Gene expression datasets for LUAD (GSE116959) and COPD (GSE76925) are available from the GEO database (https://www.ncbi.nlm.nih.gov/geo/).  Statistical Code: The R code used to perform the analyses, including Mendelian randomization, sensitivity analyses, and data visualizations, will be made publicly available upon request. The code will also be deposited in a public repository (e.g., GitHub or other open access platforms) to ensure transparency and reproducibility of the study results. |
| 20 | **Conflicts of Interest** | All authors should declare all potential conflicts of interest | **No.:**17 | The authors declare that they have no conflicts of interest related to the research, authorship, or publication of this article. There are no financial or personal relationships that could have influenced the work presented in this study. |

This checklist is copyrighted by the Equator Network under the Creative Commons Attribution 3.0 Unported (CC BY 3.0) license.

1. Skrivankova VW, Richmond RC, Woolf BAR, Yarmolinsky J, Davies NM, Swanson SA, et al. Strengthening the Reporting of Observational Studies in Epidemiology using Mendelian Randomization (STROBE-MR) Statement. JAMA. 2021;under review.

2. Skrivankova VW, Richmond RC, Woolf BAR, Davies NM, Swanson SA, VanderWeele TJ, et al. Strengthening the Reporting of Observational Studies in Epidemiology using Mendelian Randomisation (STROBE-MR): Explanation and Elaboration. BMJ. 2021;375:n2233.
